# A peptide-based PROTAC targeting FOXM1 suppresses fibrosis-associated hepatocarcinogenesis

**DOI:** 10.7150/thno.129569

**Published:** 2026-04-23

**Authors:** Dingyu Wu, Lei Duan, Di Tan, Xinyi Hua, Anping Liang, Ruiping Huai, Shanshan Qi, Zhixian Shang, Shijie Jia, Hui Qi, Xinrong Liu, Jieling Zhao, Yuhong Jiang, Rui Tan, Canquan Mao

**Affiliations:** 1 Key Laboratory of Advanced Materials Technology of the Ministry of Education, School of Life Science and Engineering, Southwest Jiaotong University, Chengdu 610031, Sichuan, China.; 2 School of Materials Science and Engineering, Southwest Jiaotong University, Chengdu 610031, Sichuan, China.; 3 School of Clinical Medicine, Dali University, Dali 671000, Yunnan, China.; 4 School of Pharmacy, Dali University, Dali 671000, Yunnan, China.

**Keywords:** Forkhead box M1 (FOXM1), proteolysis-targeting chimera (PROTAC), a disintegrin and metalloproteinase with thrombospondin motif 12 (ADAMTS12), liver fibrosis, hepatocellular carcinoma

## Abstract

**Background:**

Liver fibrosis is not only a major cause of cirrhosis but also an important risk factor for hepatocellular carcinoma (HCC). Currently, few drugs can effectively reverse established liver fibrosis. FOXM1, a transcription factor aberrantly activated in chronic liver disease, has been implicated in fibrosis-associated hepatocarcinogenesis. Nevertheless, effective pharmacological strategies for targeting FOXM1 are still lacking.

**Methods:**

We developed peptide-based proteolysis-targeting chimeras (PROTACs) by conjugating the FOXM1-binding peptide P49 with different E3 ligase ligands. Among them, P49-PROTAC^VHL^ showed the most potent FOXM1-degrading activity in HCC cells and was selected for further investigation. Its therapeutic efficacy was then evaluated in CCl_4_-induced liver fibrosis and DEN/CCl_4_-induced hepatocarcinogenesis mouse models. Transcriptome analysis was performed to elucidate the molecular mechanisms by which FOXM1 promotes fibrosis and tumor progression.

**Results:**

Mechanistically, P49-PROTAC^VHL^ recruited FOXM1 to the VHL E3 ligase, leading to its polyubiquitination and subsequent proteasomal degradation. In HCC cells, FOXM1 degradation inhibited proliferation, induced cell cycle arrest, and triggered apoptosis. In the CCl_4_ model, P49-PROTAC^VHL^ attenuated liver fibrosis, as evidenced by reduced collagen deposition, decreased α-SMA expression, and improved liver function. Mechanistic analyses, including dual-luciferase reporter assays, revealed that ADAMTS12 is a candidate transcriptional target of FOXM1. In the DEN/CCl_4_ model, P49-PROTAC^VHL^ modulated the FOXM1-ADAMTS12 axis, thereby mitigating fibrosis and suppressing hepatocarcinogenesis.

**Conclusions:**

The FOXM1-ADAMTS12 axis may represent an important molecular link between liver fibrosis and hepatocarcinogenesis. Targeting FOXM1 with peptide-based PROTACs may provide a promising therapeutic strategy to attenuate liver fibrosis and suppress HCC development.

## Introduction

Liver fibrosis is a common pathological consequence of chronic liver diseases, with major etiological factors including viral hepatitis, chronic alcohol abuse, and metabolic dysfunction-associated steatotic liver disease (MASLD). Progressive fibrogenesis disrupts hepatic architecture and function and may eventually lead to cirrhosis while increasing the risk of hepatocellular carcinoma (HCC) 1. Agents such as ursodeoxycholic acid, silybin, and obeticholic acid may improve biochemical parameters or alleviate clinical symptoms, but their antifibrotic efficacy remains limited. Although targeting TGF-β signaling holds therapeutic promise, its antifibrotic efficacy may be constrained by the stage-dependent and multifactorial nature of fibrogenesis 2. These limitations likely reflect the complexity of fibrogenesis, which involves oxidative stress, aberrant extracellular matrix (ECM) deposition, persistent inflammation, and sustained hepatic stellate cell (HSC) activation. Therefore, identifying therapeutic strategies that target key molecular drivers of fibrogenesis before the onset of cirrhosis and HCC is of critical importance.

FOXM1 regulates fundamental cellular processes, including cell cycle progression, DNA repair, and metabolic reprogramming 3. Although FOXM1 is normally expressed at low levels in differentiated adult tissues 4, its pathological reactivation in HCC is associated with tumor progression, metastasis, and poor patient survival 5. Beyond tumorigenesis, FOXM1 also contributes to pathological tissue remodeling, particularly in pulmonary and renal fibrosis 3. However, the mechanisms by which FOXM1 drives hepatic fibrogenesis and whether targeting FOXM1 holds therapeutic promise remain unclear.

Transcription factors such as FOXM1 lie at the intersection of fibrotic and oncogenic signaling networks, making them attractive therapeutic targets. FOXM1 has long been considered undruggable because it lacks a well-defined ligand-binding pocket. Although FOXM1 is a recognized therapeutic target in HCC, currently available small-molecule inhibitors, such as FDI-6 and thiostrepton, are limited by suboptimal efficacy, insufficient specificity, and off-target toxicity. In addition, the specific roles of FOXM1 in liver fibrogenesis remain poorly understood. These limitations underscore the need for novel therapeutic strategies capable of inducing potent and sustained FOXM1 degradation, thereby suppressing fibrosis-associated hepatocarcinogenesis. Proteolysis-targeting chimeras (PROTACs) have emerged as an effective strategy for selective protein degradation 6. By recruiting a protein of interest (POI) to an E3 ubiquitin ligase, PROTACs induce targeted ubiquitination followed by proteasomal degradation 7. This event-driven pharmacological modality offers advantages over conventional occupancy-driven inhibitors, particularly for proteins previously considered undruggable 8. Despite rapid advances in oncology and immunology, PROTAC technology remains largely unexplored as a therapeutic approach for chronic liver diseases, particularly in the context of fibrosis-associated hepatocarcinogenesis.

We designed a novel peptide-based PROTAC by conjugating P49, a FOXM1-binding peptide (WHDWYPEVYWR) identified through phage display screening 9, with a VHL E3 ligase ligand. This compound, termed P49-PROTAC^VHL^, efficiently and selectively degrades FOXM1. We then investigated its therapeutic potential in mouse models of liver fibrosis and fibrosis-associated hepatocarcinogenesis and explored the molecular mechanisms underlying its antifibrotic and antitumor effects.

## Materials and Methods

Detailed information on reagents and antibodies is provided in Supplementary Tables S1-S8.

### Human samples

Fibrotic liver biopsy specimens (n = 20), including 10 HBV-related and 10 MASLD-related cases, were collected from the Affiliated Hospital of Dali University. Normal liver tissues (n = 6) were obtained from archived non-diseased donor liver tissues in the Department of Pathology, Dali University. The study protocol was approved by the Ethics Committees of Dali University (Approval No. 2025-PZ-055) and Southwest Jiaotong University (Approval No. SWJTU-2403-NSFC-025). Written informed consent was obtained from all participants, and all procedures were conducted in accordance with the Declaration of Helsinki.

### Animals

Male C57BL/6J mice were obtained from Aniphe Biolaboratory (Jiangsu, China) and maintained under specific pathogen-free conditions. For the CCl_4_-induced liver fibrosis model, 8-week-old mice were used. For the DEN/CCl4-induced HCC model, male pups received a single intraperitoneal injection of diethylnitrosamine (DEN, 25 mg/kg) at postnatal day 14, followed by intraperitoneal administration of 20% CCl_4_ in olive oil beginning at week 6, as illustrated in the experimental scheme. All animal experiments were approved by the Animal Ethics Committees of Dali University and Southwest Jiaotong University.

### Synthesis of peptides and peptide-based PROTACs

Peptides were synthesized using standard Fmoc-based solid-phase peptide synthesis. Crude peptides were purified by high-performance liquid chromatography using an Agilent 1100 system and characterized by mass spectrometry on a Waters ZQ2000 instrument, with a final purity of >95%.

### Cell culture

HCCLM3, HepG2, Huh-7, HEK293T, LO-2, LX-2, and HUVEC cells were used in this study. Detailed information on these cell lines and their sources is provided in Supplementary Table S2. HCCLM3, Huh-7, HEK293T, LO-2, and LX-2 cells were cultured in high-glucose DMEM supplemented with 10% fetal bovine serum (FBS) and 1% penicillin-streptomycin. HepG2 cells were cultured in EMEM supplemented with 10% FBS and 1% penicillin-streptomycin. HUVECs were maintained in endothelial cell growth medium supplemented according to the manufacturer's instructions. All cells were cultured at 37 °C in a humidified incubator with 5% CO_2_ and were routinely tested for mycoplasma contamination.

### Cell viability and clonogenic assays

Cell viability was evaluated using a CCK-8 kit (MedChemExpress, New Jersey, USA, catalog no. HY-K0301). Absorbance at 450 nm was measured using a SpectraMax M2 microplate reader (Molecular Devices, USA), and IC_50_ values were calculated using GraphPad Prism. For the clonogenic assay, cells were treated with 3, 7, or 15 μM P49-PROTAC^VHL^ for 48 h, and then cultured in fresh medium for an additional 10-14 days. Colonies, defined as clusters containing more than 50 cells, were fixed with 4% paraformaldehyde (PFA), stained with 0.1% crystal violet, and counted using ImageJ.

### Migration, invasion, and wound healing assays

For migration assays, HCCLM3 cells (8 × 10^4^ cells/well) were seeded into the upper chambers of Transwell inserts with an 8 μm pore size (Corning, New York, USA) in 200 μL of serum-free DMEM containing 3 μM P49-PROTAC^VHL^. For invasion assays, the upper chambers were pre-coated with Matrigel, and the lower chambers were filled with 600 μL of medium containing 10% FBS. After 24 h, cells remaining on the upper surface of the membrane were removed. Cells on the lower surface were then fixed, stained, and counted.

For wound healing assays, confluent HCCLM3 cell monolayers were scratched with a pipette tip and then treated with 3 μM P49-PROTAC^VHL^ in serum-free medium. Wound images were captured at 0 and 24 h. Migration rates were calculated as [(A0 - At)/A0] × 100%, where A0 and At represent the wound area at 0 and 24 h, respectively.

### Apoptosis and cell cycle analysis

Both apoptosis and cell cycle analyses were performed by flow cytometry using a BD Accuri C6 Plus instrument, with at least 10,000 events collected per sample. For apoptosis analysis, cells were stained using an Annexin V-FITC/PI Apoptosis Detection Kit (Vazyme, Nanjing, China, catalog no. A211), and data were processed using FlowJo v10.8 software. For cell cycle analysis, cells were fixed in 75% ethanol, stained with PI (Vazyme, catalog no. AC101), and data were analyzed using ModFit LT 5.0 software.

### Cell immunofluorescence staining

Cells were fixed with 4% paraformaldehyde (PFA), permeabilized with 0.1% Triton X-100, and blocked with 5% BSA. Samples were incubated with primary antibodies overnight at 4 °C and then with fluorophore-conjugated secondary antibodies. Images were acquired using a Nikon A1+/A1R+ or a Leica TCS-SP5 confocal microscope.

### Western blotting and co-immunoprecipitation

Cell lysates were prepared in RIPA buffer supplemented with phenylmethylsulfonyl fluoride (PMSF). Proteins were separated by SDS-PAGE, transferred onto PVDF membranes, and incubated with primary antibodies, followed by HRP-conjugated secondary antibodies. Protein bands were visualized using an ECL reagent (Beyotime, Beijing, China) and imaged on an iBright FL1500 imaging system (Thermo Fisher Scientific). Band intensities were quantified using ImageJ.

For co-immunoprecipitation (Co-IP), cells were treated with P49-PROTAC^VHL^ (5, 10, or 20 μM) for 6 h and lysed. Ten percent of the lysate was saved as input, while the remaining lysate was incubated overnight at 4 °C with 3 μg of anti-FOXM1 primary antibody or 3 μg of an isotype-matched IgG control (Servicebio, Wuhan, China). Protein A/G magnetic beads were then added and incubated for 8 h, followed by washing and immunoblot analysis of the eluates.

### Microscale thermophoresis

pCMV-EGFP-FOXM1 and pCMV-EGFP plasmids were transfected into HEK293T cells. After 48 h, cell lysates containing EGFP-labeled proteins were collected for microscale thermophoresis (MST) analysis. Lysates were mixed at a 1:1 ratio with 2-fold serial dilutions of P49-PROTAC^VHL^ (16 concentrations in total) and incubated for 5 min at room temperature in the dark. MST measurements were performed using a Monolith NT.115 instrument (NanoTemper Technologies, Germany). Dissociation constants (*K_d_*) were calculated using MO.Affinity Analysis software (NanoTemper Technologies) based on the law of mass action.

### Cellular thermal shift assay

HCCLM3 cells were treated with 10 μM P49-PROTAC^VHL^ for 12 h and then harvested and lysed in IP lysis buffer (ABclonal, Wuhan, China). The lysates were equally divided and heated at the indicated temperatures (48-63 °C) for 3 min in a LongGene A200 gradient thermal cycler (Hangzhou, China), after which they were allowed to cool to room temperature for an additional 3 min. The samples were then centrifuged at 20,000 × g for 20 min at 4 °C to isolate the soluble protein fractions. The levels of soluble FOXM1 were then assessed by immunoblotting.

### Bimolecular fluorescence complementation

*FOXM1* and *VHL* were subcloned into pcDNA3.1-YCE (VC155) and pcDNA3.1-YNE (VN173), respectively. HEK293T cells were co-transfected with *FOXM1*-YCE and *VHL*-YNE plasmids. After 48 h, the cells were treated with 4 μM P49-PROTAC^VHL^ or vehicle for 6 h. Reconstituted Venus fluorescence was visualized using a Nikon A1+/A1R+ confocal microscope.

### Cycloheximide chase assay

HCCLM3 cells were treated with 50 μg/mL cycloheximide (CHX) alone or in combination with 10 μM P49-PROTAC^VHL^ for the indicated times (0, 1, 2, 4, 6, and 8 h). At each time point, cells were harvested and lysed in RIPA buffer supplemented with protease inhibitors. Equal amounts of protein were separated by SDS-PAGE and immunoblotted with an anti-FOXM1 antibody.

### Quantitative real-time PCR

Total RNA was extracted from liver tissues or cultured cells using a FastPure RNA extraction kit (Vazyme). RNA concentration and purity were assessed using a NanoDrop 2000 spectrophotometer (Thermo Fisher). cDNA was synthesized from 1 μg of total RNA using the HiScript IV RT SuperMix kit (Vazyme). qRT-PCR was performed using Taq Pro SYBR Green Master Mix (Vazyme) on a StepOnePlus Real-Time PCR system (Thermo Fisher). Relative expression levels were normalized to GAPDH and calculated using the 2^-ΔΔCt^ method. Primer sequences are provided in Supplementary Table S4.

### Dual-luciferase reporter assay

The *ADAMTS12* promoter fragment (-2.0 kb) was cloned into the pGL3.1-Basic vector (Miaoling, Wuhan, China), and *FOXM1* was cloned into the pcDNA3.1 vector (Miaoling). HEK293T cells (2 × 10^5^ cells/well in 24-well plates) were co-transfected with 1 μg of pcDNA3.1-*FOXM1*, 1 μg of the pGL3.1-*ADAMTS12* promoter reporter, and 0.1 μg of pRL-TK as an internal Renilla luciferase control. After 48 h, firefly and Renilla luciferase activities were measured using a Dual-Luciferase Reporter Assay Kit (GeneCreate, Wuhan, China) on a Varioskan LUX microplate reader (Thermo Fisher). Promoter activity was expressed as the ratio of firefly luciferase activity to Renilla luciferase activity.

### RNA sequencing and data analysis

Liver tissues from the CCl_4_ + DMSO group and the CCl_4_ + P49-PROTAC^VHL^ group (n = 3 per group) were snap-frozen in liquid nitrogen, and total RNA was extracted using TRIzol reagent (Thermo Fisher). RNA concentration and purity were assessed using a NanoDrop 2000 spectrophotometer (Thermo Fisher). RNA sequencing was performed by Shanghai Personal Biotechnology Co., Ltd. on an Illumina NovaSeq 6000 platform. Differentially expressed genes (DEGs) were identified using DESeq2 in R, with an adjusted P value < 0.05 and |log2 fold change| > 1 as the thresholds. Gene Ontology (GO) and Kyoto Encyclopedia of Genes and Genomes (KEGG) pathway enrichment analyses were performed to investigate the biological functions and signaling pathways associated with the DEGs.

### CCl_4_-induced liver fibrosis model

Mice were randomly assigned to five groups (n = 6 per group): (i) olive oil + DMSO, (ii) olive oil + P49-PROTAC^VHL^, (iii) CCl_4_ + DMSO, (iv) CCl_4_ + silybin, and (v) CCl_4_ + P49-PROTAC^VHL^. Liver fibrosis was induced by intraperitoneal injection of 20% CCl_4_ in olive oil (2 μL/g body weight) twice weekly for 8 weeks. Starting from week 6, mice received daily intraperitoneal administration of P49-PROTAC^VHL^ or silybin at 15 mg/kg.

### DEN/CCl_4_-induced HCC model

Mice were randomly divided into four groups (n = 10 per group): (i) control, mice received diethylnitrosamine (DEN) followed by olive oil administration; (ii) model, mice received DEN followed by CCl_4_ administration; (iii) early intervention, mice received DEN and CCl_4_ and were treated with P49-PROTAC^VHL^ starting at week 16; and (iv) treatment, mice received DEN and CCl_4_ and were treated with P49-PROTAC^VHL^ starting at week 22. Fourteen-day-old mice received a single intraperitoneal injection of DEN (25 mg/kg). Beginning at week 6, mice in the model, early intervention, and treatment groups were intraperitoneally injected with 20% CCl_4_ in olive oil (2 μL/g body weight) twice weekly, whereas mice in the control group received olive oil alone. P49-PROTAC^VHL^ (15 mg/kg, i.p.) was administered daily from week 16 to week 24 in the early intervention group and from week 22 to week 24 in the treatment group. All mice were sacrificed at week 24, and liver tissues were collected for subsequent analyses. The number of visible tumor nodules on the liver surface was recorded, and tumor size was measured at necropsy. Final sample sizes at the endpoint were smaller because some mice died during DEN/CCl4 model induction.

### Flow cytometric analysis

Fresh liver single-cell suspensions were prepared using a mouse tumor tissue dissociation kit (Absin, catalog no. abs50090). Cells (5-10 × 10^6^/mL) were resuspended in staining buffer (BD Pharmingen, catalog no. 554656), blocked with an anti-CD16/CD32 monoclonal antibody (Fc block), and stained with a fluorophore-conjugated antibody cocktail for 30 min at 4 °C in the dark. Samples were analyzed using an Agilent NovoCyte Advanteon flow cytometer with the following gating strategy: single cells → viable cells (7-AAD^-^) → CD45^+^ cells. Immune subpopulations were identified using FlowJo v10.8 software with the following markers: T cells (CD3^+^ NK1.1^-^), NK cells (CD3^-^ NK1.1^+^), dendritic cells (CD11c^+^ I-A/I-E^+^), Kupffer cells (CD11b^+^ F4/80^+^), and monocytes (CD11b^+^ F4/80^-^).

### ELISA assay

Serum samples were centrifuged at 3,000 × g for 15 min at 4 °C and stored at -80 °C until analysis. Liver tissues (approximately 100 mg) were homogenized in PBS supplemented with protease inhibitors and centrifuged at 12,000 × g for 20 min at 4 °C to collect the supernatants. Serum and liver tissue supernatants were analyzed using commercial assay kits according to the manufacturers' instructions. Absorbance was measured at 450 nm with a reference wavelength of 570 nm using a Varioskan LUX microplate reader.

### Histological assessment of liver fibrosis

Paraffin-embedded liver sections (4-5 μm thick) were deparaffinized and subjected to histological staining to evaluate collagen deposition. Sections were stained with either a Masson's Trichrome Staining Kit (Solarbio, Beijing, China, catalog no. G1340) or a Sirius Red Staining Kit (Solarbio, catalog no. G1472) according to the manufacturer's instructions. Stained tissue sections were imaged using an Olympus BX53 light microscope. Fibrotic and collagen-positive areas in randomly selected fields were quantified as percentages of the total tissue area using Fiji (ImageJ) software.

### Immunohistochemistry

Paraffin-embedded sections were deparaffinized and subjected to antigen retrieval in citrate buffer (pH 6.0, 90-100 °C for 15 min). After blocking with 5% bovine serum albumin (BSA) in TBST, the sections were incubated with primary antibodies overnight at 4 °C, followed by incubation with HRP-conjugated secondary antibodies for 1 h at room temperature. Immunoreactivity was developed using a DAB chromogenic substrate (Solarbio, catalog no. G1212), and the sections were counterstained with hematoxylin and mounted with neutral balsam.

### Multiplex immunofluorescence staining

Formalin-fixed, paraffin-embedded tissue sections were subjected to sequential multiplex staining using a TSA Plus reagent kit (Servicebio, catalog no. G1257) according to the manufacturer's instructions. After deparaffinization, antigen retrieval was performed in citrate or EDTA buffer, as appropriate. Sections were sequentially incubated with primary antibodies against albumin, CD11b, CD31, α-SMA, ADAMTS12, and FOXM1, followed by HRP-conjugated secondary antibodies and TSA fluorophore labeling. Between staining rounds, antibody complexes were stripped by microwave treatment in citrate buffer. Nuclei were counterstained with DAPI, and images were acquired using a Leica TCS-SP5 confocal microscope. Detailed antibody information is provided in Supplementary Table S1.

### Fluorescence *in situ* hybridization

Under RNase-free conditions, liver tissues were fixed, dehydrated, cleared in xylene, and embedded in paraffin. Sections were deparaffinized, rehydrated through a graded ethanol series, and treated with proteinase K (20 μg/mL) at 37 °C for 15 min. After prehybridization, the sections were hybridized overnight at 37 °C with A*damts12*-specific probes (Servicebio; sequences provided in Supplementary Table S6). Hybridization signals were detected using a Cy3 FISH detection kit (Servicebio, catalog no. GF002) according to the manufacturer's instructions. Images were acquired using a Nikon ECLIPSE Ci-FL fluorescence microscope equipped with a Cy3 filter set (excitation 550 nm, emission 570 nm).

### Tube formation assay

HUVECs were transfected with *FOXM1* or *ADAMTS12* expression plasmids, or with *ADAMTS12* siRNA, using Lipomaster 3000 Transfection Reagent (Vazyme). After 48 h, cells were harvested and seeded onto Matrigel-precoated 24-well plates at a density of 8 × 10^4^ cells/well in medium containing 5 μM P49-PROTAC^VHL^. After 24 h of incubation, cells were stained with 2 μM Calcein-AM (Servicebio) for 30 min at 37 °C. Tube formation was quantified using the Angiogenesis Analyzer plugin in ImageJ by measuring the total tube length and the number of nodes.

### Scanning electron microscopy

Livers were perfusion-fixed with 2.5% glutaraldehyde in 0.1 M phosphate buffer (pH 7.4), trimmed into blocks (<5 mm^3^), and immersed in the same fixative at 4 °C for at least 4 h. After postfixation with 1% osmium tetroxide at 4 °C for 2 h in the dark, samples were washed in phosphate buffer, dehydrated through a graded ethanol series, critical point dried using a Quorum K850, and sputter-coated with gold (approximately 10 nm thick) using a Hitachi MC1000. Images were acquired using a Hitachi SU8100 scanning electron microscope.

### Transmission electron microscopy

Liver tissues were fixed in 2.5% glutaraldehyde in 0.1 M phosphate buffer (pH 7.4) at 4 °C for 4 h, postfixed in 1% osmium tetroxide at 4 °C for 2 h, dehydrated through a graded ethanol series, and embedded in Poly/Bed 812 epoxy resin (Ted Pella, catalog no. 18109). Ultrathin sections (60 nm) were stained with uranyl acetate and lead citrate. Images were acquired using a Hitachi HT7800 transmission electron microscope.

### Statistical analysis

Data are presented as the mean ± SD from at least three independent biological replicates. Statistical significance between two groups was assessed using an unpaired Student's t-test. Comparisons among more than two groups were performed using one-way ANOVA followed by Tukey's multiple comparisons test. *P value < 0.05* was considered statistically significant. All statistical analyses were performed using GraphPad Prism 9.5.1.

## Results

### Rational design of peptide-based PROTACs identifies P49-PROTAC^VHL^ as the lead FOXM1 degrader

**Figure 1:**
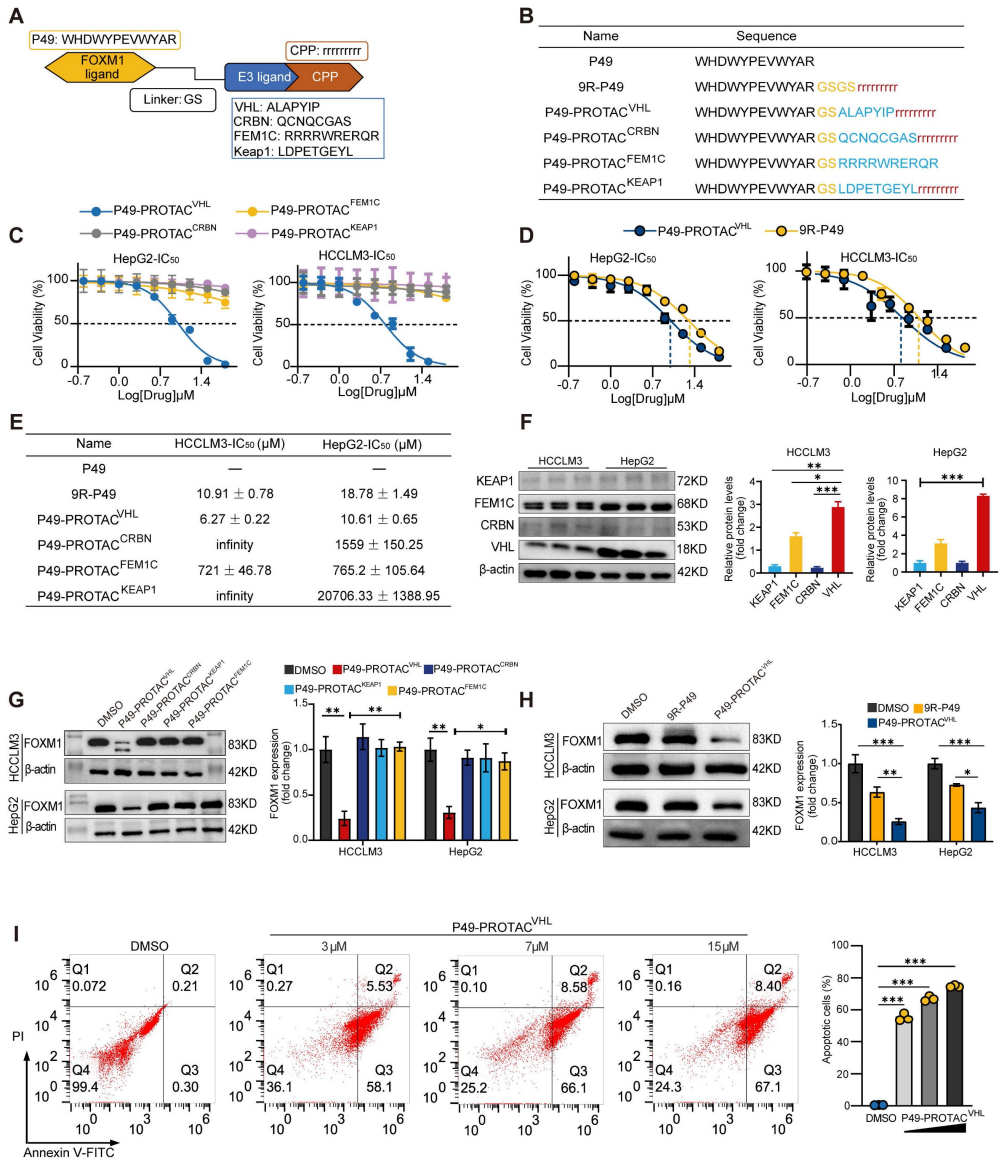
** Design and screening of FOXM1**-**targeting peptide PROTACs. (A)** Schematic of FOXM1-targeting peptide PROTACs consisting of P49, a Gly-Ser linker, and distinct E3 ligase ligands. A nine-D-arginine cell-penetrating peptide (9R) was used in the VHL-, CRBN-, and KEAP1-recruiting constructs.** (B)** Amino acid sequences of the synthesized peptide constructs.** (C)** CCK-8 analysis of HCCLM3 and HepG2 cells treated with the indicated PROTACs for 24 h (n = 5).** (D)** CCK-8 analysis of HCCLM3 and HepG2 cells treated with P49-PROTAC^VHL^ or 9R-P49 for 24 h (n = 5).** (E)** Calculated IC50 values.** (F)** Western blot analysis of KEAP1, FEM1C, CRBN, and VHL in HCCLM3 and HepG2 cells (n = 3).** (G)** Western blot screening of FOXM1 degradation after treatment with each PROTAC at 5 μM for 24 h (n = 3).** (H)** Western blot analysis of FOXM1 after treatment with P49-PROTAC^VHL^ or 9R-P49 at 5 μM for 24 h (n = 3).** (I)** Flow cytometric analysis of apoptosis after P49-PROTAC^VHL^ treatment for 24 h. Bar graphs show total apoptotic cells (n = 3). Data are mean ± SD. *P < 0.05, **P < 0.01, ***P < 0.001.

A series of FOXM1-targeting peptide-based PROTACs was rationally designed and synthesized, using the previously identified FOXM1-binding peptide P49 9 as the targeting warhead. Each chimera comprised the P49 peptide linked via a flexible glycine-serine (GS) linker to a previously reported E3 ligase-binding moiety targeting VHL 10, CRBN 11, KEAP1 12, or FEM1C 13 (Figure 1A). A C-terminal nona-arginine (9R) cell-penetrating peptide was added to the VHL-, CRBN-, and KEAP1-recruiting constructs to facilitate intracellular delivery. In contrast, the FEM1C-based construct was designed without an additional cell-penetrating sequence, as the arginine-rich motif RRRRWRERQR has previously been shown to facilitate cellular uptake 14 (Figure 1B).

Endogenous FOXM1 expression was profiled in five liver-derived cell lines (HCCLM3, Huh-7, HepG2, LX-2, and LO-2) to identify a suitable cell model for degrader evaluation. Immunoblotting showed that HCCLM3 and HepG2 cells exhibited the highest basal FOXM1 protein levels (Figure S1A). Initial phenotypic screening using CCK-8 assays identified the VHL-recruiting chimera P49-PROTAC^VHL^ as the lead candidate. This compound displayed strong antiproliferative effects, with IC_50_ values of 6.27 ± 0.22 μM in HCCLM3 cells and 10.61 ± 0.65 μM in HepG2 cells, whereas the parental 9R-P49 peptide showed substantially weaker activity (Figure 1C-E). To further investigate the superior activity of P49-PROTAC^VHL^, the endogenous expression levels of the recruited E3 ligases (VHL, CRBN, KEAP1, and FEM1C) were examined in HCCLM3 and HepG2 cells. Among them, VHL was expressed at relatively high levels in both cell lines (Figure 1F), which may contribute to the enhanced activity of the VHL-based degrader. P49-PROTAC^VHL^ (5 μM, 24 h) markedly reduced FOXM1 protein levels in both HCCLM3 and HepG2 cells (Figure 1G, H). In contrast, the parental 9R-P49 peptide and the other E3-recruiting constructs (CRBN-, KEAP1-, and FEM1C-based) exhibited limited FOXM1 degradation, indicating that appropriate E3 ligase selection is critical for potent and selective target depletion.

Functional analyses in HCCLM3 cells further showed that P49-PROTAC^VHL^ exerted potent antitumor effects, including dose-dependent induction of apoptosis (Figure 1I) and marked inhibition of colony formation, migration, and invasion (Figure S1B-E). Flow cytometry demonstrated cell cycle arrest in the G0/G1 phase (Figure S1F), and qRT-PCR revealed significant downregulation of the canonical FOXM1 target genes *CDC25B* and *CCNB1* (Figure S1G).

### P49-PROTAC^VHL^ induces FOXM1 degradation through the VHL-dependent ubiquitin-proteasome pathway

**Figure 2:**
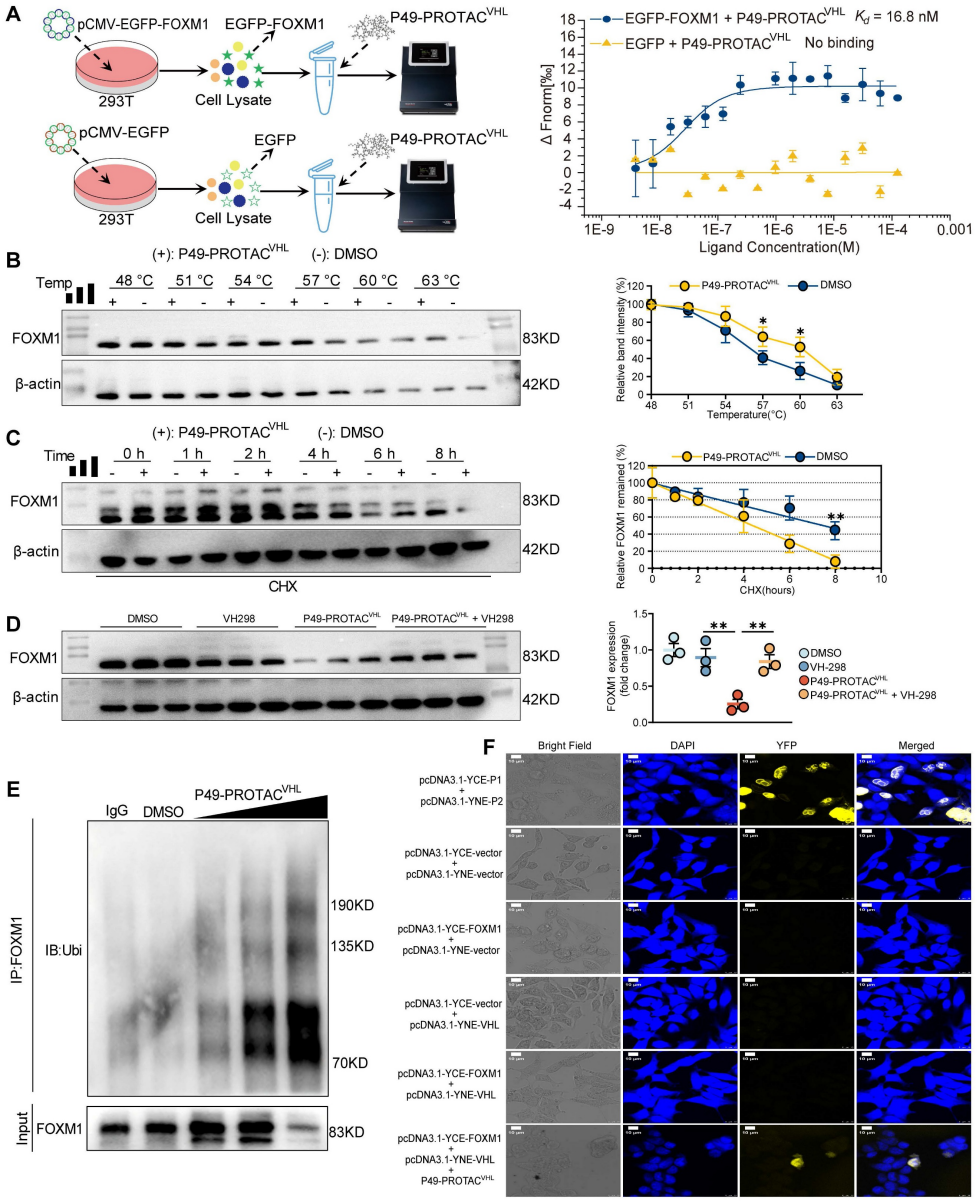
** P49-PROTAC^VHL^ promotes VHL-dependent ubiquitination and degradation of FOXM1. (A)** Lysate-based MST analysis of P49-PROTAC^VHL^ binding to EGFP-FOXM1. EGFP alone was used as a negative control. **(B)** CETSA of FOXM1 in HCCLM3 cells treated with P49-PROTAC^VHL^ (10 μM) or vehicle (n = 3). **(C)** CHX chase assay of FOXM1 in HCCLM3 cells treated with P49-PROTAC^VHL^ (10 μM) or DMSO (n = 3). **(D)** Western blot analysis of FOXM1 in HCCLM3 cells pretreated with the VHL ligand VH298 before P49-PROTAC^VHL^ exposure (n = 3). **(E)** Co-IP assay of FOXM1 ubiquitination in HCCLM3 cells treated with P49-PROTAC^VHL^. FOXM1 was immunoprecipitated and ubiquitin was detected by immunoblotting (n = 3). **(F)** BiFC assay of FOXM1-VHL interaction in HEK293T cells. Reconstituted YFP fluorescence indicates PROTAC-induced FOXM1-VHL association. A_2_A_R_ homodimerization (P1 + P2) was used as a positive control. Scale bar, 10 μm. Data are mean ± SD. *P < 0.05, **P < 0.01, ***P < 0.001.

Microscale thermophoresis (MST) demonstrated that P49-PROTAC^VHL^ specifically binds to FOXM1. In lysates containing EGFP-tagged FOXM1, P49-PROTAC^VHL^ bound FOXM1 with a *K_d_* of 16.8 nM, whereas no binding was detected with EGFP alone, excluding interference from the EGFP tag (Figure 2A). CETSA further supported intracellular target engagement by showing that P49-PROTAC^VHL^ increased the thermal stability of endogenous FOXM1 in HCCLM3 cells. In the absence of the compound, FOXM1 became progressively unstable as the temperature increased; however, P49-PROTAC^VHL^ significantly delayed FOXM1 destabilization, particularly between 57 °C and 60 °C (Figure 2B). Conversely, CHX chase assays showed that, when *de novo* protein synthesis was blocked, P49-PROTAC^VHL^ markedly accelerated FOXM1 degradation, resulting in an approximately 90% reduction in FOXM1 protein levels within 8 h, whereas more than 40% of FOXM1 remained in vehicle-treated cells (Figure 2C).

The role of the VHL-ubiquitin-proteasome pathway in P49-PROTAC^VHL^-induced FOXM1 degradation was evaluated in HCCLM3 cells using VH298, a competitive VHL ligand. VH298 effectively abrogated P49-PROTAC^VHL^-induced FOXM1 degradation, demonstrating that this process requires VHL engagement (Figure 2D). Co-immunoprecipitation assays revealed that P49-PROTAC^VHL^ markedly enhanced FOXM1 ubiquitination, as detected by immunoblotting for ubiquitin following FOXM1 immunoprecipitation (Figure 2E). Consistently, cellular immunofluorescence analysis with co-staining for FOXM1 (green) and ubiquitin (red) showed increased colocalization after P49-PROTAC^VHL^ treatment in HCCLM3 cells, further supporting enhanced FOXM1 ubiquitination (Figure S2A).

Bimolecular fluorescence complementation (BiFC) assays confirmed that P49-PROTAC^VHL^ induced ternary complex formation between FOXM1 and VHL. Co-expression of FOXM1 and VHL alone yielded little to no fluorescence signal; however, addition of P49-PROTAC^VHL^ produced a strong BiFC signal, indicating that the PROTAC promoted the interaction between the two proteins by inducing ternary complex formation (Figure 2F). In addition, a FITC-labeled P49-PROTAC^VHL^ analog was used to examine its intracellular association with FOXM1 or VHL in HEK293T cells separately expressing FOXM1-mCherry or VHL-turboRFP. The FITC-labeled PROTAC was efficiently internalized and showed clear colocalization with FOXM1 and VHL, respectively, supporting its ability to engage both proteins in cells (Figure S2B).

### FOXM1 is upregulated in liver fibrosis, and P49-PROTAC^VHL^ suppresses hepatic stellate cell activation

**Figure 3:**
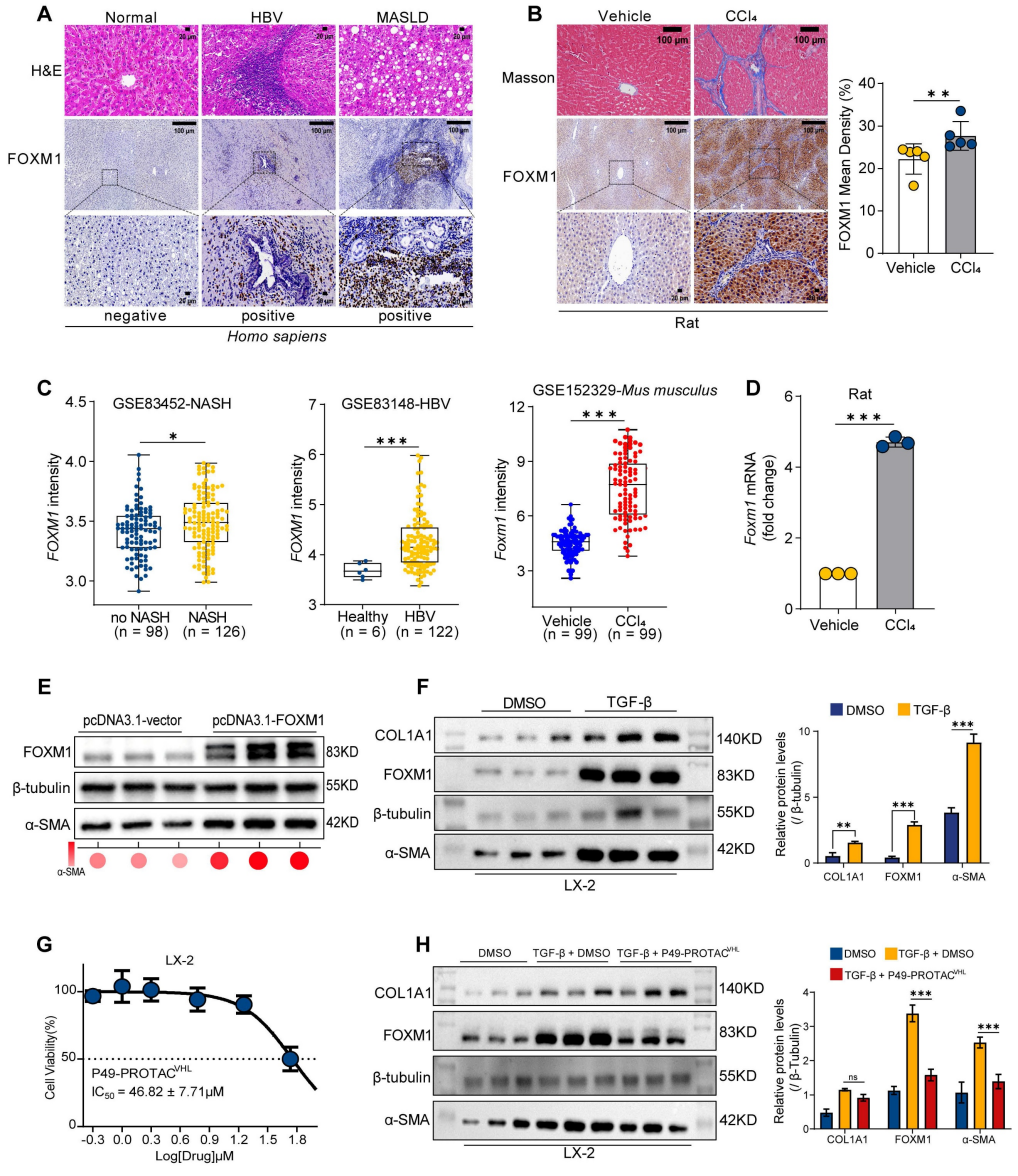
** FOXM1 expression in fibrotic liver tissue and its relationship to hepatic stellate cell activation. (A)** Representative H&E staining and FOXM1 immunohistochemistry in liver sections from normal donor tissues (n = 6), HBV-related fibrosis (n = 10), and MASLD-related fibrosis (n = 10). Scale bars, 100 μm (top) and 20 μm (bottom).** (B)** Masson's trichrome staining and FOXM1 immunohistochemistry in liver sections from a previously established CCl_4_-induced rat fibrosis model and vehicle controls (n = 5).** (C)** Analysis of FOXM1 expression in fibrosis datasets.** (D)** qRT-PCR analysis of *Foxm1* mRNA in fibrotic rat livers (n = 3).** (E)** Western blot analysis of FOXM1 and α-SMA in LX-2 cells transfected with pcDNA3.1-FOXM1 or empty vector (n = 3).** (F)** Western blot analysis of FOXM1, COL1A1, and α-SMA in LX-2 cells treated with TGF-β1 (10 ng/mL, 24 h; n = 3).** (G)** CCK-8 analysis of P49-PROTAC^VHL^ in LX-2 cells (n = 3).** (H)** Western blot analysis of FOXM1 and α-SMA in TGF-β1-stimulated LX-2 cells treated with 5 μM P49-PROTAC^VHL^ (n = 3). Data are mean ± SD. *P < 0.05, **P < 0.01, ***P < 0.001.

FOXM1 expression was analyzed in human fibrotic liver specimens, fibrosis-related datasets, and experimental models to investigate its association with liver fibrosis. Immunohistochemical analysis showed marked FOXM1 expression in the fibrotic liver tissues from patients with hepatitis B virus (HBV) infection or MASLD, whereas little or no signal was detected in healthy controls (Figure 3A). FOXM1 upregulation was also observed in archived liver samples from a previously established CCl_4_-induced rat fibrosis model (Figure 3B). Analysis of Gene Expression Omnibus (GEO) datasets further confirmed increased *FOXM1* expression across multiple human (HBV- and MASLD-related) and murine fibrosis cohorts (Figure 3C). Consistently, *Foxm1* mRNA levels were elevated in the same rat fibrotic liver tissues (Figure 3D), supporting FOXM1 as a conserved factor associated with liver fibrogenesis.

The effect of P49-PROTAC^VHL^ on hepatic stellate cell (HSC) activation was then evaluated. FOXM1 overexpression in LX-2 cells increased the expression of the fibrogenic marker α-SMA (Figure 3E). In addition, TGF-β1 stimulation induced concomitant upregulation of FOXM1, COL1A1, and α-SMA (Figure 3F). To exclude nonspecific cytotoxicity as the primary cause of these effects, cell viability assays were performed and showed that P49-PROTAC^VHL^ exhibited an IC_50_ of 46.82 ± 7.71 μM in LX-2 cells (Figure 3G). Notably, at a non-cytotoxic concentration (5 μM), P49-PROTAC^VHL^ significantly reduced TGF-β1-induced FOXM1 and α-SMA protein expression, indicating suppression of HSC activation under non-cytotoxic conditions (Figure 3H).

To further assess whether the cellular effects of P49-PROTAC^VHL^ were associated with FOXM1 targeting rather than nonspecific cytotoxicity, we assessed its target dependence and degradation selectivity. In HEK293T cells, P49-PROTAC^VHL^ exhibited greater cytotoxic activity in FOXM1-overexpressing cells than in wild-type controls, supporting a FOXM1-dependent cellular response (Figure S3A). In addition, to evaluate degradation selectivity, we examined closely related FOX family members in HCCLM3 cells. P49-PROTAC^VHL^ induced dose-dependent degradation of FOXM1, whereas the protein levels of the homologous transcription factors FOXA1 and FOXO1 remained unchanged (Figure S3B). Together, these findings indicate that P49-PROTAC^VHL^ selectively degrades FOXM1 over the tested FOX family members.

### P49-PROTAC^VHL^ ameliorates liver fibrosis in mice through FOXM1 targeting

**Figure 4:**
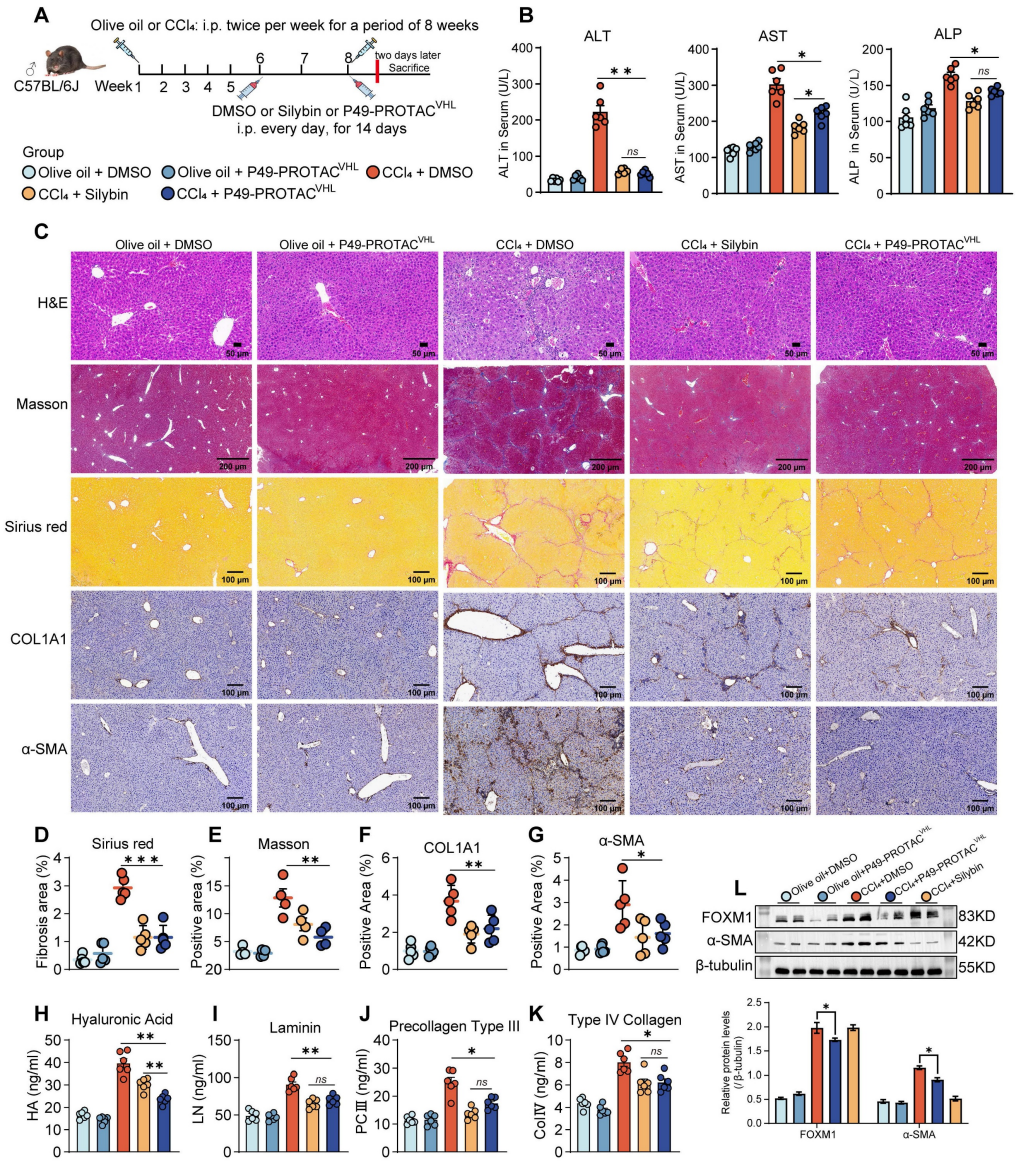
** P49-PROTAC^VHL^ attenuates liver fibrosis in CCl_4_-induced mice. (A)** Experimental schedule of the CCl_4_-induced liver fibrosis model and treatment regimen (n = 6 per group). **(B)** Serum ALT, AST, and ALP levels (n = 6). **(C)** Representative liver images showing H&E, Masson's trichrome, Sirius Red, and immunohistochemistry for COL1A1 and α-SMA. Scale bars, 50 μm for H&E and 200 μm for the remaining panels. **(D-G)** Quantification of Sirius Red-positive area (n = 5), Masson-positive area (n = 4), COL1A1-positive area (n = 5), and α-SMA-positive area (n = 5). **(H-K)** Serum hyaluronic acid, laminin, procollagen type III, and type IV collagen levels (n = 6). **(L)** Western blot analysis of FOXM1 and α-SMA in liver lysates (n = 3). Data are mean ± SD. *P < 0.05, **P < 0.01, ***P < 0.001.

The therapeutic efficacy of P49-PROTAC^VHL^ was assessed in a CCl_4_-induced mouse model of liver fibrosis (Figure 4A). CCl_4_ administration caused marked hepatomegaly and increased the liver-to-body weight ratio (Figure S4A, B). P49-PROTAC^VHL^ treatment significantly alleviated liver injury, as reflected by reduced serum ALT, AST, and ALP levels (Figure 4B) and decreased hepatic hydroxyproline content (Figure S4C).

Histopathological analyses showed that P49-PROTAC^VHL^ treatment improved hepatic architecture and reduced necrosis and inflammatory infiltration, with an overall histological improvement similar to that observed with silybin, a known antifibrotic agent (Figure 4C). Sirius Red and Masson's trichrome staining further confirmed a significant reduction in collagen deposition in the CCl_4_ + P49-PROTAC^VHL^ group (Figure 4D, E). IHC analysis demonstrated markedly reduced expression of the fibrotic markers COL1A1 and α-SMA in the treatment group compared with the fibrotic control group (Figure 4F, G).

Serological analysis showed that P49-PROTAC^VHL^ reduced serum fibrosis-related markers, including type IV collagen, procollagen type III, laminin, and hyaluronic acid (Figure 4H-K). The treatment also attenuated hepatic oxidative stress, as indicated by increased SOD and CAT activities, elevated GSH levels, and reduced MDA accumulation (Figure S4D-G). In parallel, P49-PROTAC^VHL^ exerted immunomodulatory effects by decreasing the proinflammatory cytokines TNF-α, IL-6, and IL-1β while increasing the anti-inflammatory cytokine IL-10 (Figure S4H-K). Immunoblotting showed marked reductions in FOXM1 and α-SMA protein expression in the CCl_4_ + P49-PROTAC^VHL^ group (Figure 4L). At the transcript level, P49-PROTAC^VHL^ significantly reduced *Col1a1* and *Acta2* mRNA expression, whereas Tgfb1 expression remained unchanged (Figure S4L).

Importantly, P49-PROTAC^VHL^ was well tolerated under the tested conditions. No histopathological lesions were detected in the lungs or kidneys of treated mice (Figure S4M). Moreover, 20 days of P49-PROTAC^VHL^ administration in normal mice did not affect renal function markers, including BUN, creatinine, and uric acid, compared with vehicle-treated controls (Figure S4N).

### FOXM1 transcriptionally regulates *ADAMTS12* and is associated with ECM remodeling and sinusoidal capillarization

**Figure 5:**
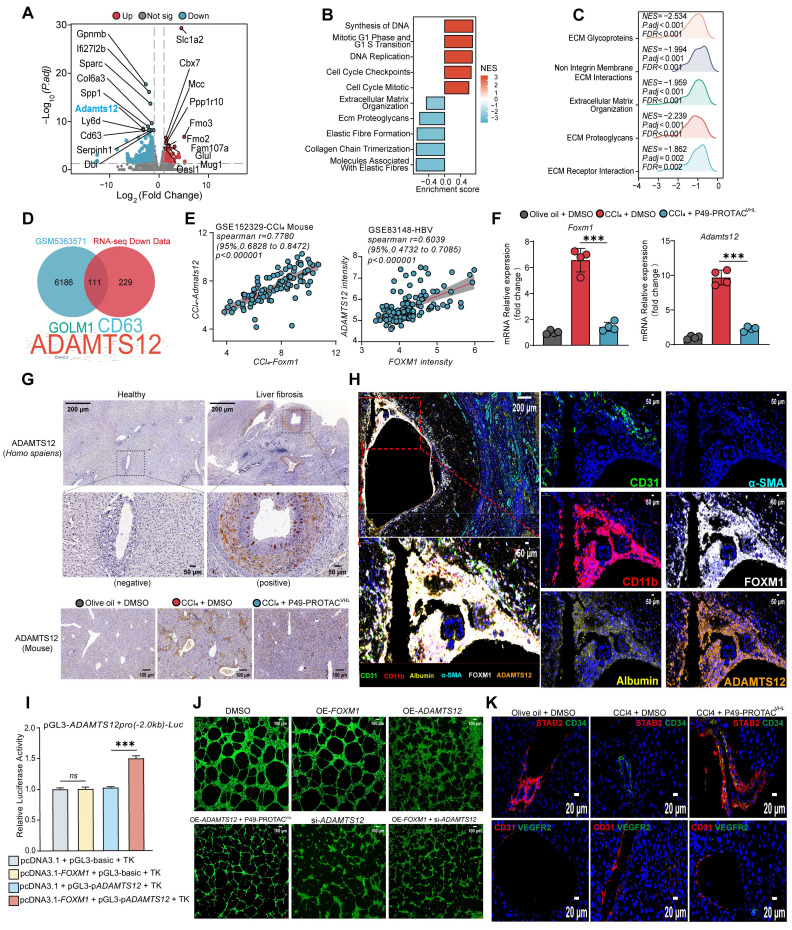
** FOXM1 regulates ADAMTS12 expression and is associated with ECM remodeling and LSEC capillarization. (A)** Volcano plot of RNA-seq data from CCl_4_ + DMSO and CCl_4_ + P49-PROTAC^VHL^ groups.** (B-C)** Pathway enrichment analysis and GSEA.** (D)** Overlap of FOXM1 ChIP-seq targets (GSM5363571) with genes downregulated by P49-PROTAC^VHL^.** (E)** Correlation analysis of *FOXM1* and *ADAMTS12* expression in fibrosis datasets.** (F)** qRT-PCR analysis of *Foxm1* and *Adamts12* mRNA in mouse livers (n = 4).** (G)** ADAMTS12 immunohistochemistry in human liver tissues and fibrotic mouse livers. Scale bars: 200 μm and 50 μm for human samples, and 100 μm for mouse samples.** (H)** Multiplex immunofluorescence analysis of FOXM1, ADAMTS12, CD31, CD11b, albumin, and α-SMA in human fibrotic liver tissues. Scale bars, 200 μm and 50 μm.** (I)** Dual-luciferase reporter assay of *ADAMTS12* promoter activity (n = 3).** (J)** HUVEC tube formation assay (n = 4). Scale bar, 100 μm. **(K)** Immunofluorescence analysis of STAB2, CD31, CD34, and VEGFR2 in mouse livers (n = 3). Scale bar, 20 μm. Data are mean ± SD. *P < 0.05, **P < 0.01, ***P < 0.001.

RNA sequencing (RNA-seq) was performed on liver tissues from the CCl_4_ + DMSO and CCl_4_ + P49-PROTAC^VHL^ groups, and the resulting transcriptomic data were further subjected to transcription factor enrichment analysis (Figure S5A, B). Differential expression analysis identified 91 upregulated and 340 downregulated genes (Figure S5C). Volcano plot analysis revealed marked downregulation of several extracellular matrix (ECM)-related genes, including *Gpnmb*, *Adamts12*, *Spp1*,* Col6a3*, and *Serpinh1* (Figure 5A). Gene Set Enrichment Analysis (GSEA) further demonstrated negative enrichment of ECM organization and ECM-receptor interaction pathways in the P49-PROTAC^VHL^-treated group (Figure 5B, C).

Integration of the RNA-seq data with a publicly available FOXM1 ChIP-seq dataset (GSM5363571) identified ADAMTS12 as a candidate transcriptional target of FOXM1 (Figure 5D). ADAMTS12 is a metalloprotease implicated in extracellular matrix (ECM) remodeling 15 and stromal remodeling in chronic liver disease 16. ADAMTS12 expression was significantly reduced after P49-PROTAC^VHL^ treatment, and its transcript levels showed a strong positive correlation with FOXM1 across multiple independent clinical liver fibrosis cohorts (Figure 5E, Figure S5D). In the fibrotic mouse model, P49-PROTAC^VHL^ treatment also reduced the transcript levels of both *Foxm1* and *Adamts12* (Figure 5F). In addition, IHC analysis showed reduced ADAMTS12 protein expression in the fibrotic liver tissues after P49-PROTAC^VHL^ treatment (Figure 5G, Figure S5E).

Multiplex immunofluorescence showed that ADAMTS12 was predominantly localized to the perivascular region and partially colocalized with the endothelial marker CD31. Areas with intense FOXM1 staining also exhibited stronger ADAMTS12 signals, indicating spatial association within the fibrotic niche (Figure 5H). P49-PROTAC^VHL^ treatment also significantly reduced the expression of several ECM-related genes, including *Spp1*, *Col3a1*, *Col4a1*, *Timp1*, and *Fn1*, whereas *Lamb1* expression remained unchanged (Figure S5F). Fibronectin deposition was likewise markedly reduced after P49-PROTAC^VHL^ treatment (Figure S5G).

Luciferase reporter assays showed that FOXM1 enhanced ADAMTS12 promoter activity (Figure 5I). P49-PROTAC^VHL^ treatment suppressed this activation and also reduced the transcriptional activity of the canonical FOXM1 target gene PLK1 (Figure S5H). The role of ADAMTS12 in FOXM1-related endothelial phenotypes was further examined using rescue experiments in the HUVEC tube formation assay. FOXM1 overexpression enhanced tube formation, whereas this effect was abolished by ADAMTS12 knockdown. Conversely, ectopic ADAMTS12 expression reversed the anti-angiogenic effect of P49-PROTAC^VHL^ (Figure 5J, Figure S5I), suggesting that ADAMTS12 contributes to FOXM1-related angiogenic responses.

Phenotypic alterations in liver sinusoidal endothelial cells (LSECs) were then examined because ADAMTS12 was associated with vascular changes. Under physiological conditions, differentiated LSECs maintain a specialized phenotype characterized by low or absent CD34 expression and expression of scavenger receptors such as STAB2 17, 18. During fibrogenesis, however, LSECs undergo capillarization, a pathological phenotypic switch associated with loss of specialized LSEC features, including STAB2 expression, and aberrant induction of CD34 19. Immunofluorescence analysis showed that P49-PROTAC^VHL^ attenuated LSEC capillarization and preserved the STAB2^high^ and CD34^low^ phenotype, whereas vehicle-treated fibrotic controls displayed a pathological STAB2^low^ and CD34^high^ phenotype (Figure 5K). P49-PROTAC^VHL^ reduced CD31-positive area, whereas VEGFR2 expression remained unchanged (Figure 5K, Figure S5J). In addition, vWF staining was reduced after treatment (Figure S5K). Together, these findings indicate that P49-PROTAC^VHL^ alleviates pathological sinusoidal remodeling during liver fibrosis.

### P49-PROTAC^VHL^ attenuates liver fibrosis and tumor burden in a DEN/CCl_4_ murine model

**Figure 6:**
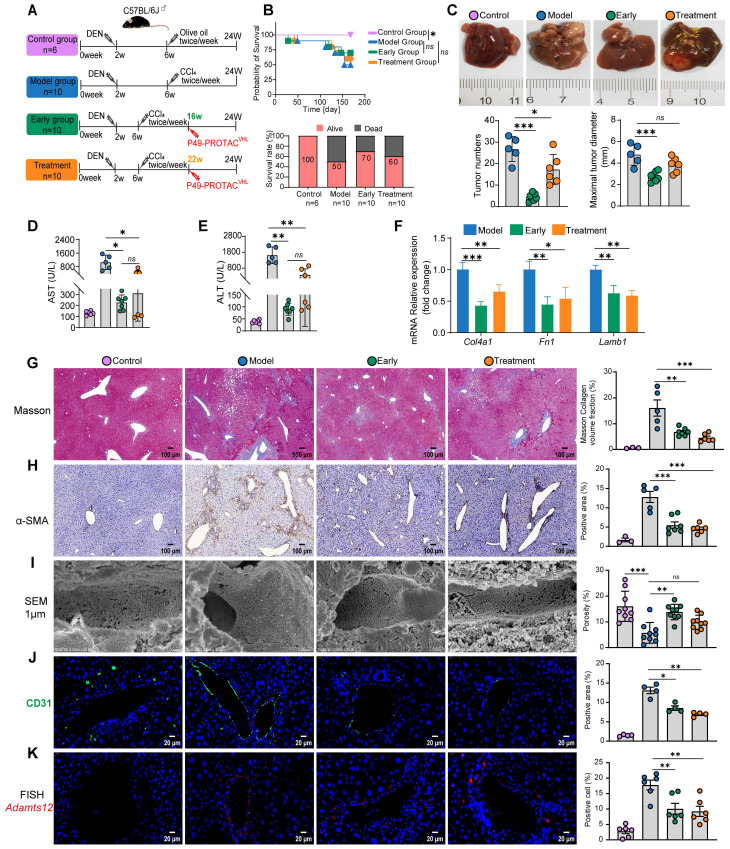
** P49-PROTAC^VHL^ suppresses hepatocarcinogenesis in DEN/CCl_4_-induced HCC mice. (A)** Experimental design. **(B)** Kaplan-Meier survival analysis. **(C)** Gross liver images with quantification of tumor number and maximal diameter. **(D-E)** Serum ALT and AST levels (Control, n = 6; Model, n = 5; Early, n = 7; Treatment, n = 6). The numbers shown represent the mice available for endpoint analysis; some mice died during DEN/CCl4 model induction. **(F)** qRT-PCR analysis of *Col4a1*, *Fn1*, and Lamb1 in liver tumors (n = 3). **(G)** Masson's trichrome staining with fibrotic area quantification. Scale bar, 100 μm (Control, n = 3; Model, n = 5; Early, n = 7; Treatment, n = 6). **(H)** α-SMA IHC with positive area quantification. Scale bar, 100 μm (Control, n = 3; Model, n = 5; Early, n = 7; Treatment, n = 6). **(I)** SEM images showing sinusoidal ultrastructure and quantification of sinusoidal porosity. Scale bar, 1 μm (three fields per sample, n = 3). **(J)** CD31 immunofluorescence with positive area quantification. Scale bar, 20 μm (n = 3). **(K)** FISH detection of *Adamts12* mRNA and quantification of positive cells. Scale bar, 20 μm (two fields per sample, n = 3). Data are mean ± SD. *P < 0.05, **P < 0.01, ***P < 0.001.

The efficacy of P49-PROTAC^VHL^ was evaluated in a DEN/CCl_4_-induced mouse model of fibrosis-associated hepatocarcinogenesis. Because this model recapitulates the pathological progression from liver fibrosis to hepatocellular carcinoma, we established two treatment schedules: an early intervention group beginning at week 16 and a late treatment group beginning at week 22 (Figure 6A). Liver ultrasonography at week 22 confirmed the presence of macroscopic nodular lesions in all DEN/CCl_4_-treated groups (Figure S6A). No significant differences in overall survival were observed among the groups during the study period (Figure 6B), whereas tumor burden varied markedly.

P49-PROTAC^VHL^ showed antitumor activity in both the early intervention and late treatment groups, with a greater reduction in tumor burden in the early intervention group. Early intervention significantly reduced tumor volume, nodule multiplicity, absolute liver weight, and the liver-to-body weight ratio relative to vehicle-treated controls (Figure 6C, Figure S6B). H&E staining showed disrupted hepatic architecture and hepatocellular necrosis in the model group, whereas P49-PROTAC^VHL^ treatment alleviated these histopathological changes (Figure S6C). In both the early and late treatment groups, Ki-67 staining showed reduced tumor cell proliferation (Figure S6D).

P49-PROTAC^VHL^ attenuated hepatic fibrosis in both the early intervention and late treatment groups. Both treatment regimens significantly reduced ALT and AST levels, consistent with reduced hepatocellular injury (Figure 6D, E). Gene expression analysis showed reduced expression of fibrosis-associated extracellular matrix genes, including *Col4a1*, *Fn1*, and *Lamb1* (Figure 6F). Histopathological findings were consistent with these results: Masson's trichrome and Sirius Red staining both showed reduced collagen accumulation (Figure 6G, Figure S6E), and immunohistochemistry confirmed decreased α-SMA and COL1A1 expression (Figure 6H, Figure S6F).

Vascular remodeling within the tumor microenvironment was further evaluated by TEM, SEM, and CD31 immunofluorescence. Transmission electron microscopy (TEM) showed that P49-PROTAC^VHL^ attenuated sinusoidal capillarization and pathological basement membrane formation (Figure S6G). Scanning electron microscopy (SEM) showed significant preservation of sinusoidal porosity (fenestrations) in the early intervention group (Figure 6I). CD31 immunofluorescence staining also showed reduced abnormal vascular remodeling after P49-PROTAC^VHL^ treatment (Figure 6J).

Similar to the findings in the fibrosis model, fluorescence *in situ* hybridization (FISH) showed that P49-PROTAC^VHL^ treatment significantly reduced the perivascular *Adamts12* mRNA signal in tumor tissue (Figure 6K), further linking the FOXM1-ADAMTS12 axis to pathological vascular remodeling. Because endothelial-to-mesenchymal transition (EndMT) has been associated with pathological angiogenesis, mesenchymal-associated markers were next examined in tumor tissues. Immunoblotting showed that P49-PROTAC^VHL^ treatment significantly reduced FOXM1 protein expression, accompanied by a marked decrease in the mesenchymal marker N-cadherin. In contrast, E-cadherin expression showed an upward trend but did not reach statistical significance (Figure S6H).

**Figure 7:**
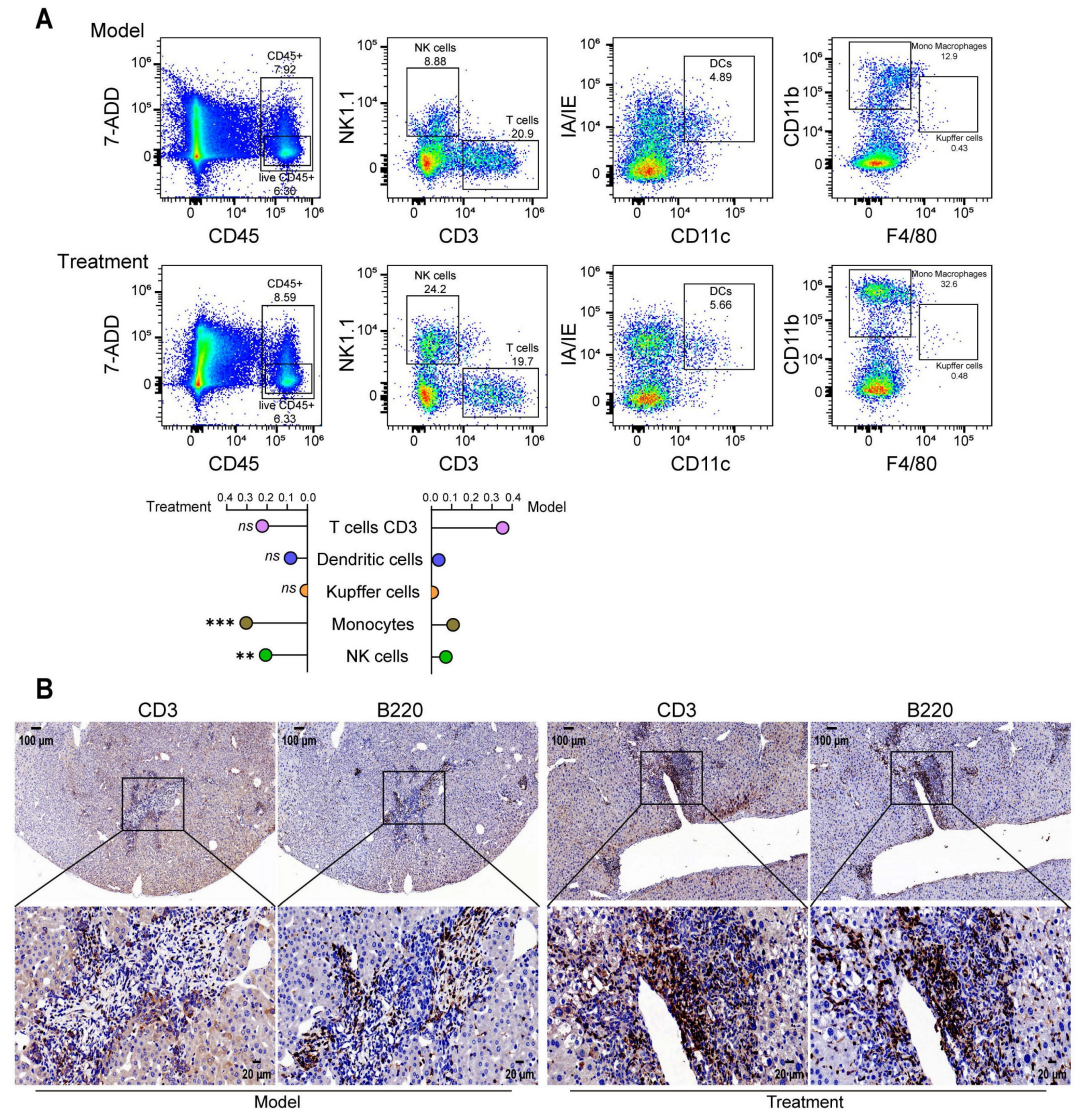
** P49-PROTAC^VHL^ remodels the tumor immune microenvironment in DEN/CCl_4_-induced HCC mice. (A)** Flow cytometric analysis of hepatic immune subsets: T cells (CD3^+^ NK1.1^-^), dendritic cells (CD11c^+^ I-A/I-E^+^), Kupffer cells (CD11b^+^ F4/80^+^), monocytes (CD11b^+^ F4/80^-^), and NK cells (CD3^-^ NK1.1^+^) (n = 3).** (B)** Immunohistochemistry for CD3 (T cells) and B220 (B cells) in serial sections. Scale bar, 50 μm. Data are mean ± SD. *P < 0.05, **P < 0.01, ***P < 0.001.

P49-PROTAC^VHL^ modulated the tumor immune microenvironment. Flow cytometric analysis showed increased infiltration of monocytes and natural killer (NK) cells in the late treatment group (Figure 7A). In addition, serial liver sections separately stained for the T-cell marker CD3 and the B-cell marker B220 showed clustered lymphoid aggregates composed of both T and B cells at hepatic tumor sites after P49-PROTAC^VHL^ treatment, suggesting the presence of putative immature TLS-like aggregates. These aggregates were not observed in untreated controls (Figure 7B).

Together, these findings indicate that P49-PROTAC^VHL^ attenuates fibrosis-associated hepatocarcinogenesis, suppresses tumor growth, and mitigates pathological vascular and immune alterations within the tumor microenvironment.

## Discussion

Fibrosis-associated liver cancer remains difficult to treat because current therapeutic strategies are unable to effectively interrupt the progression from chronic liver disease to malignancy. FOXM1, a transcription factor, may drive the progression of fibrosis-associated hepatocarcinogenesis by linking hepatic stellate cell activation to HCC progression. Previous studies have linked FOXM1 to CCL2-dependent macrophage recruitment 20, the FOXM1/CMA/ER stress axis in nonalcoholic steatohepatitis 21, and transcriptional regulation of MAT2A/MAT2B 22. In this study, we developed a peptide-based PROTAC, P49-PROTAC^VHL^, to target FOXM1 for degradation. Our data show that FOXM1 degradation alleviates liver fibrosis and suppresses fibrosis-associated hepatocarcinogenesis, supporting targeted protein degradation as a strategy to simultaneously mitigate fibrogenesis and tumor progression. FOXM1 was consistently upregulated in the fibrotic liver tissues from patients with HBV-related disease and MASLD, in experimental fibrosis models, and in TGF-β1-stimulated HSCs. These findings support a broader role for FOXM1 in fibrogenic remodeling, including extracellular matrix accumulation and matrix reorganization.

Our data also showed that cellular sensitivity to P49-PROTAC^VHL^ varied across cell types and was associated with endogenous FOXM1 abundance. The degrader inhibited proliferation in FOXM1-high HCCLM3 and HepG2 cells while exerting limited effects on LX-2 cells at concentrations used to assess antifibrotic activity (Figure S1A). This pattern is consistent with FOXM1-dependent activity rather than nonspecific cytotoxicity. Importantly, the antifibrotic effect of P49-PROTAC^VHL^ is unlikely to result from direct toxicity to stromal cells. Instead, under non-cytotoxic conditions, the degrader suppressed activation-associated fibrotic markers in HSCs, including COL1A1 and α-SMA. These findings support the therapeutic potential of FOXM1-targeted degradation in fibrosis-associated liver disease.

Mechanistic analyses support a role for the FOXM1-ADAMTS12 axis in extracellular matrix remodeling and sinusoidal vascular alterations. Integration of RNA-seq data with a public FOXM1 ChIP-seq dataset, together with luciferase reporter assays, identified *ADAMTS12* as a candidate transcriptional target of FOXM1. ADAMTS12, a secreted metalloproteinase, has previously been implicated in ECM remodeling, angiogenesis, and cerebrovascular disease-related processes 23. In our study, FOXM1 degradation reduced ADAMTS12 expression and was accompanied by downregulation of ECM-related genes, including *Col4a1*, *Fn1,* and *Spp1*, as well as attenuation of microvascular abnormalities. P49-PROTAC^VHL^ also alleviated liver sinusoidal endothelial cell capillarization, a hallmark of early fibrotic remodeling characterized by loss of fenestrations and basement membrane deposition. Under physiological conditions, LSECs maintain a specialized differentiated phenotype characterized by high STAB2 and low CD34 expression 24, whereas this phenotype is progressively lost during capillarization in liver disease 25. FOXM1 has been implicated in endothelial regeneration 26 and in proliferative vascular remodeling, particularly through promoting vascular smooth muscle cell proliferation 27, 28. Together, these data suggest that FOXM1 contributes to pathological ECM remodeling and sinusoidal vascular dysfunction, at least in part through regulation of ADAMTS12.

In the DEN/CCl_4_ model, treatment was initiated at week 16 to assess early intervention during the fibrosis-to-HCC transition. Because no pathological or ultrasonographic examination was performed at that time point, we cannot determine whether microscopic tumor foci had already formed before treatment initiation. The reduced tumor burden observed in the early intervention group supports an attenuating effect on fibrosis-associated hepatocarcinogenesis, but does not prove a truly preventive effect. In addition, despite significantly reducing tumor burden (Figure 6C) and Ki-67 positivity (Figure S6D), P49-PROTAC^VHL^ did not significantly improve overall survival (Figure 6B). Several factors may account for this discrepancy. The DEN/CCl_4_ model reproduces necroinflammation, fibrosis, cirrhosis, and HCC, but its utility for survival analysis is limited by the cumulative toxicity of the inducing agents 29, 30. In advanced stages, mortality is likely driven not only by tumor burden but also by decompensated cirrhosis and liver failure. Under these conditions, a 14-day treatment period may be sufficient to reduce tumor burden without being long enough to translate into a measurable survival benefit. In addition, the limited follow-up, low event rate, and modest sample size reduced the power of the survival analysis. Because this study was designed primarily to evaluate FOXM1 targeting during the fibrosis-to-HCC transition rather than survival outcomes, future studies with larger cohorts and longer observation periods will be required to determine whether the antitumor effects of P49-PROTAC^VHL^ can ultimately improve survival.

Most preclinical models do not fully capture the genetic, microenvironmental, and metabolic complexity of human HCC, and model selection should therefore be aligned with the specific translational question being addressed. The DEN/CCl_4_ model enabled evaluation of FOXM1-targeted intervention in fibrosis-associated hepatocarcinogenesis, but it does not fully recapitulate the heterogeneity of human disease. Further validation in models that more closely reflect human pathophysiology will be important for clinical translation. Patient-derived organoid (PDO) and patient-derived xenograft (PDX) models may provide useful next steps, as they preserve key aspects of tumor heterogeneity and may facilitate further evaluation of FOXM1-targeted therapeutic strategies.

Recent applications of PROTAC technology in FOXM1-targeted cancer therapy include a degrader that suppresses glucose metabolism and PD-L1 expression through FOXM1 degradation 31, as well as a self-assembling peptide-based PROTAC prodrug (NFTP) with integrin α6-mediated targeting and tumor microenvironment-responsive release 32. In contrast, the present study was based on a FOXM1-binding peptide identified in our laboratory, followed by systematic evaluation of multiple E3 ligase-recruiting strategies to identify the most effective degrader design. We also improved intracellular delivery by incorporating a cell-penetrating sequence. Importantly, this work extends FOXM1-targeted degradation from conventional tumor suppression to the fibrosis-associated carcinogenic process, thereby providing a framework for intervention across the continuum of disease progression. Our findings also underscore several principles relevant to PROTAC design. Target abundance in the selected disease model is an important determinant of response, as illustrated by the greater activity observed in FOXM1-high HCCLM3 and HepG2 cells. In addition, PROTAC efficacy depends on productive ternary complex formation, which is influenced both by compatibility between the target and the recruited E3 ligase and by endogenous ligase expression. Although the human genome encodes hundreds of E3 ligases 33, only a limited number have been validated for targeted protein degradation, emphasizing the importance of rational E3 ligase selection 34.

Peptide-based PROTACs offer substantial therapeutic promise, but several challenges remain, including protease sensitivity, limited stability, rapid renal clearance, and uneven tissue distribution. Future efforts should focus on conformationally constrained peptide designs, such as hydrocarbon stapling, double-stapled architectures, and β-hairpin engineering, to improve structural preorganization, intracellular stability, and resistance to degradation 35-38, and on advanced formulations, including lipid-like nanoparticles 39, 40 and self-assembled nanoplatforms 41, 42, to enhance bioavailability and tissue delivery. Equally important, comprehensive pharmacokinetic and biodistribution studies will be needed to define and further optimize the in vivo behavior of these peptide-based degraders.

## Conclusions

Our findings suggest that the FOXM1-ADAMTS12 axis may represent an important molecular link between liver fibrosis and hepatocarcinogenesis. We show that the peptide-based PROTAC P49-PROTAC^VHL^ induces FOXM1 degradation through the VHL-dependent ubiquitin-proteasome pathway, thereby exerting both antifibrotic and antitumor effects. In preclinical models, FOXM1 degradation inhibited hepatic stellate cell activation, attenuated pathological extracellular matrix remodeling, alleviated sinusoidal capillarization, and reduced tumor burden. Together, these findings support FOXM1-targeted protein degradation as a promising therapeutic strategy for fibrosis-associated liver disease and provide a rationale for the further development of FOXM1-directed PROTACs.

## Supplementary Material

Supplementary figures and tables.

## Abbreviations

ECM: extracellular matrix
HSC: hepatic stellate cell
MST: microscale thermophoresis
CETSA: cellular thermal shift assay
CHX: cycloheximide
IHC: immunohistochemistry
Co-IP: co-immunoprecipitation
HBV: hepatitis B virus
MASLD: metabolic dysfunction-associated steatotic liver disease
GEO: Gene Expression Omnibus
GSEA: gene set enrichment analysis
LSEC: liver sinusoidal endothelial cell
TLS: tertiary lymphoid structure
FISH: fluorescence *in situ* hybridization
EMT: epithelial-mesenchymal transition
SEM: scanning electron microscopy
TEM: transmission electron microscopy
IF: immunofluorescence
BiFC: bimolecular fluorescence complementation
qRT-PCR: quantitative real-time PCR
