# Supplementary material for: A peptide-based PROTAC targeting FOXM1 suppresses fibrosis-associated hepatocarcinogenesis
Source: Theranostics. 2026 Apr 23;16(11):6266–84. doi: 10.7150/thno.129569 (PMC13142673; doi:10.7150/thno.129569)
Supplement: Supplementary file 1 — Supplementary figures and tables. [file thnov16p6266s1.pdf]

Supporting Information

A peptide-based PROTAC targeting FOXM1 suppresses fibrosis-associated hepatocarcinogenesis

Dingyu Wu[1,2], Lei Duan[3], Di Tan[4], Xinyi Hua[1,2], Anping Liang[1,2], Ruiping Huai[1,2], Shanshan Qi[1,2], Zhixian Shang[1], Shijie Jia[1], Hui Qi[1], Xinrong Liu[1], Jieling Zhao[1], Yuhong Jiang[1], Rui Tan[1,*], Canquan Mao[1,*]

[1] Key Laboratory of Advanced Materials Technology of the Ministry of Education, School of Life Science and Engineering, Southwest Jiaotong University, Chengdu 610031, Sichuan, China.

[2] School of Materials Science and Engineering, Southwest Jiaotong University, Chengdu 610031, Sichuan, China.

[3] School of Clinical Medicine, Dali University, Dali 671000, Yunnan, China.

[4] School of Pharmacy, Dali University, Dali 671000, Yunnan, China.

***Corresponding authors**

Rui Tan, tanrui@swjtu.edu.cn

Canquan Mao, maocq@swjtu.edu.cn

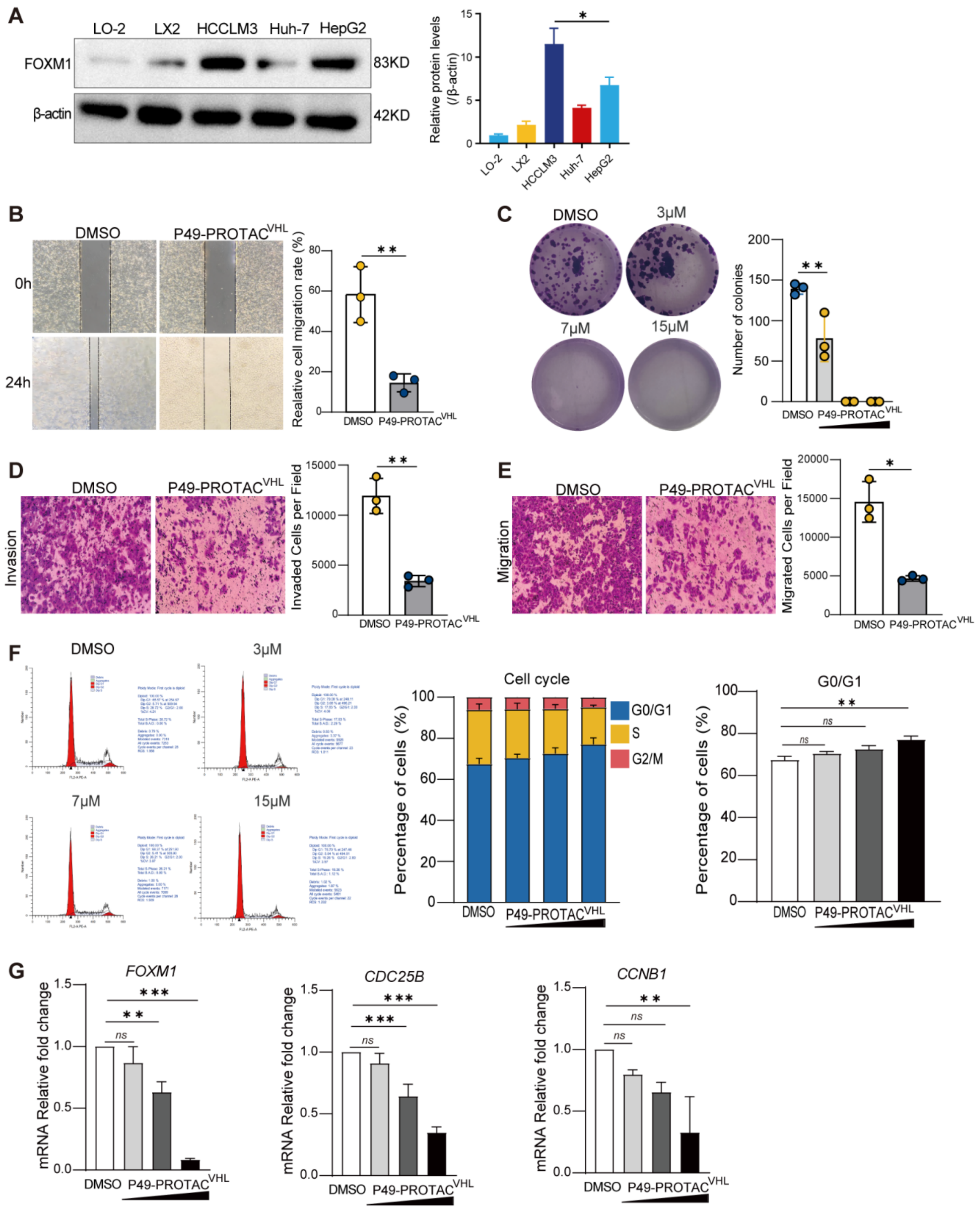

Figure S1. **(A)** Western blot analysis of baseline FOXM1 in liver-derived cell lines (n = 3). **(B)** Wound healing assay after treatment with P49-PROTAC^{VHL} (3 μM, 24 h; n = 3). **(C)** Colony formation assay after treatment with P49-PROTAC^{VHL} (3, 7, and 15 μM; n = 3). **(D–E)** Transwell invasion (D) and migration (E) assays after treatment with P49-PROTAC^{VHL} (3 μM; n = 3). **(F)** Flow cytometric analysis of cell cycle distribution after P49-PROTAC^{VHL} treatment (n = 3). **(G)** qRT-PCR analysis of *FOXM1*, *CDC25B*, and *CCNB1* after treatment with P49-PROTAC^{VHL} (3, 7, and 15 μM, 24 h; n = 3). Data are mean ± SD. **P < 0.05, **P < 0.01, ***P < 0.001.*

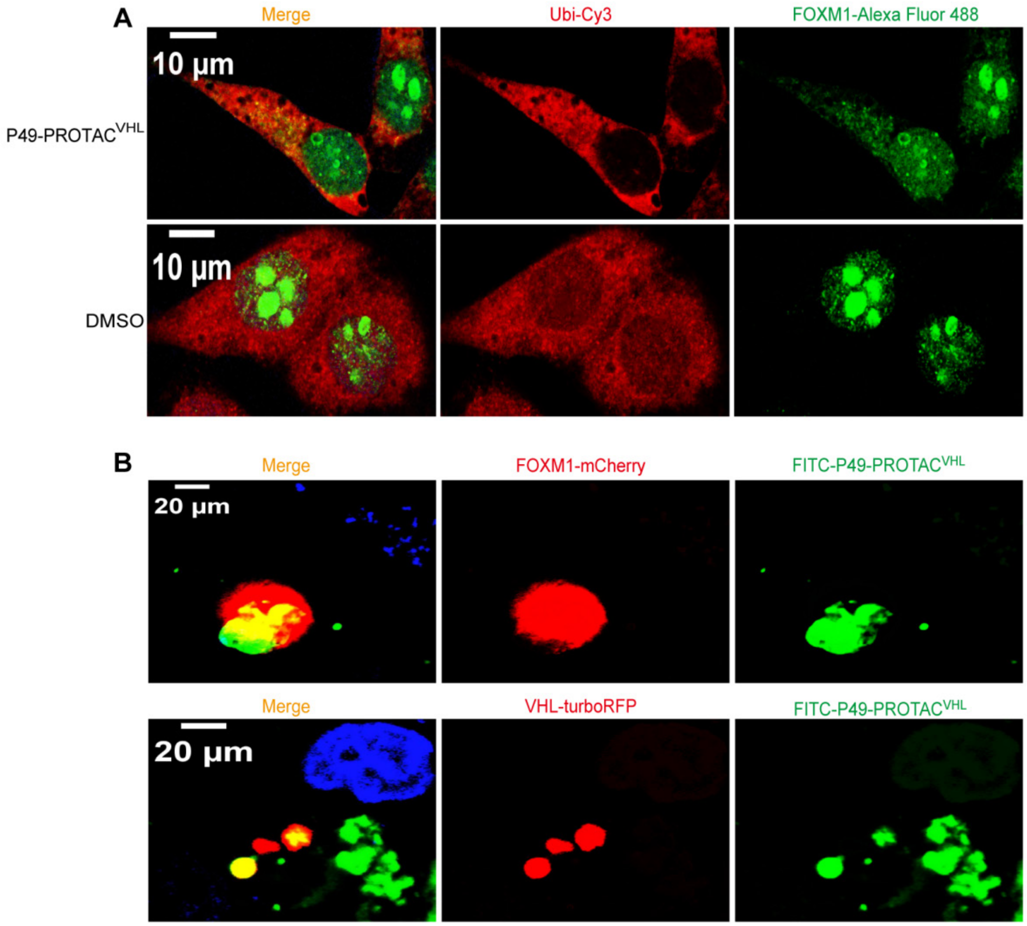

Figure S2. (A) Immunofluorescence staining of FOXM1 (green) and ubiquitin (red) in HCCLM3 cells treated with P49-PROTAC^{VHL} (4 μM, 6 h) or DMSO. Merged images are shown. Scale bar, 10 μm. **(B)** Confocal images of HEK293T cells expressing FOXM1-mCherry (top) or VHL-turboRFP (bottom) after incubation with FITC-labeled P49-PROTAC^{VHL} (green). Merged images are shown. Scale bar, 20 μm.

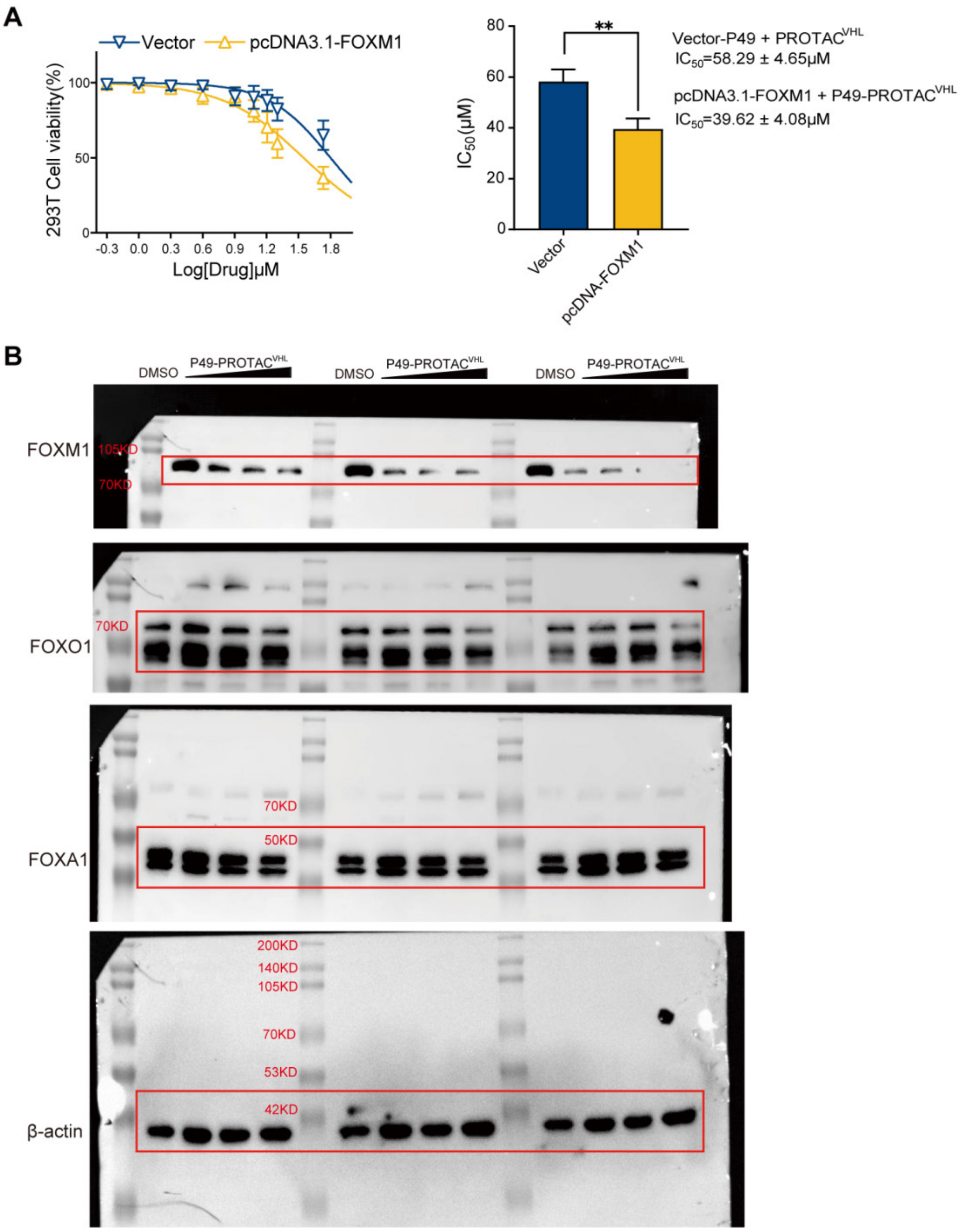

Figure S3. **(A)** CCK-8 analysis of HEK293T cells transfected with pcDNA3.1-FOXM1 or empty vector (n = 3). **(B)** Western blot analysis of FOXM1, FOXO1, and FOXA1 in HCCLM3 cells treated with P49-PROTAC^{VHL} (3, 7, and 15 μM, 24 h; n = 3). Data are mean ± SD. **P < 0.05, **P < 0.01, ***P < 0.001*.

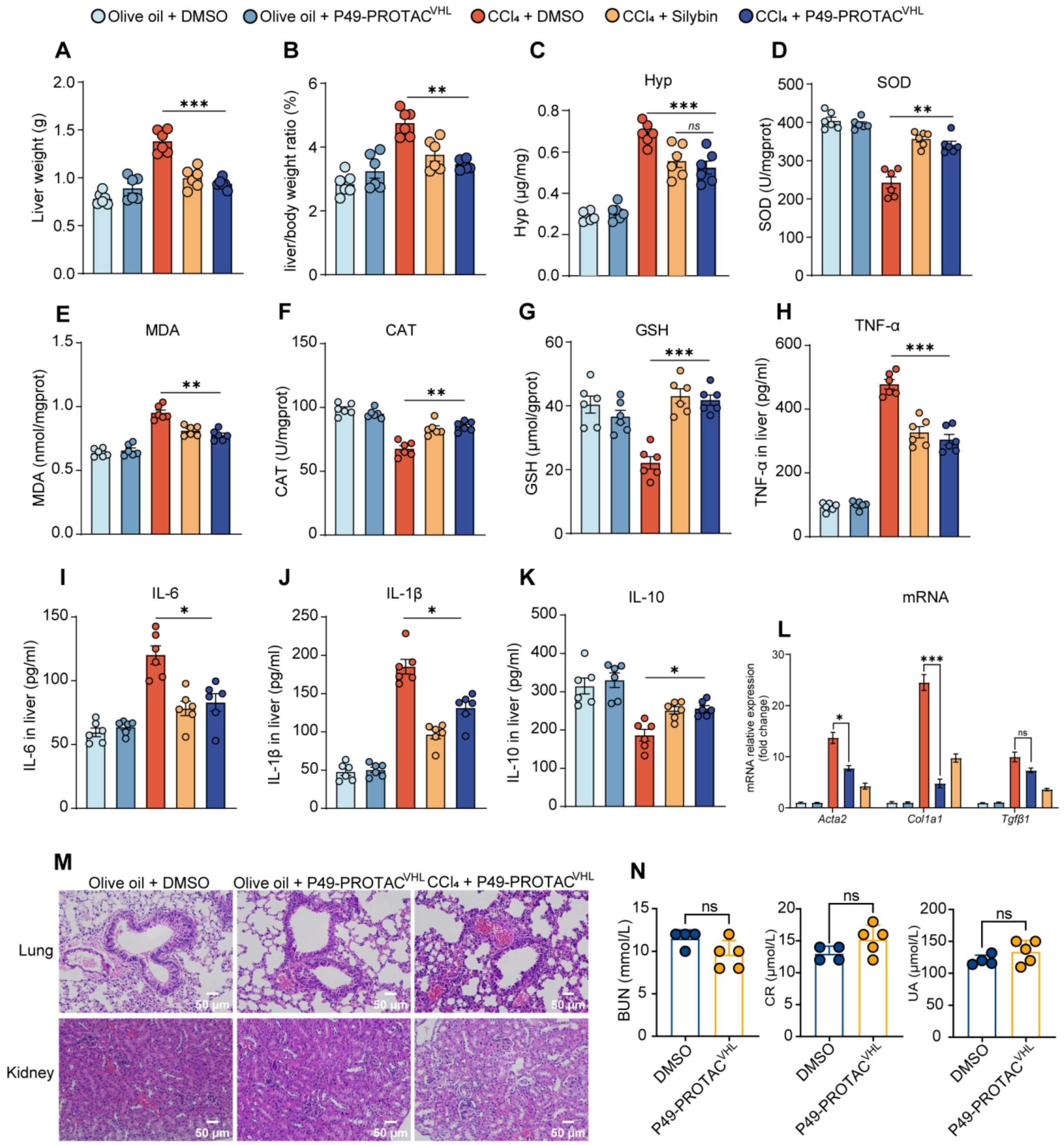

Figure S4. (A–B) Liver weight and liver index (liver-to-body weight ratio) (n = 6). **(C)** Hepatic hydroxyproline content (n = 6). **(D–G)** Hepatic SOD, MDA, CAT, and GSH levels (n = 6). **(H–K)** ELISA analysis of TNF-α, IL-6, IL-1β, and IL-10 in liver tissues (n = 6). **(L)** qRT-PCR analysis of *Acta2*, *Col1a1*, and *Tgfβ1* mRNA expression (n = 3). **(M)** Representative H&E staining of lung and kidney sections. Scale bar, 50 µm. **(N)** Serum BUN, creatinine (CR), and uric acid (UA) levels in C57BL/6J mice after repeated administration of P49-PROTAC^{VHL}. Vehicle, n = 4; P49-PROTAC^{VHL}, n = 5. Data are mean ± SD. **P < 0.05, **P < 0.01, ***P < 0.001*.

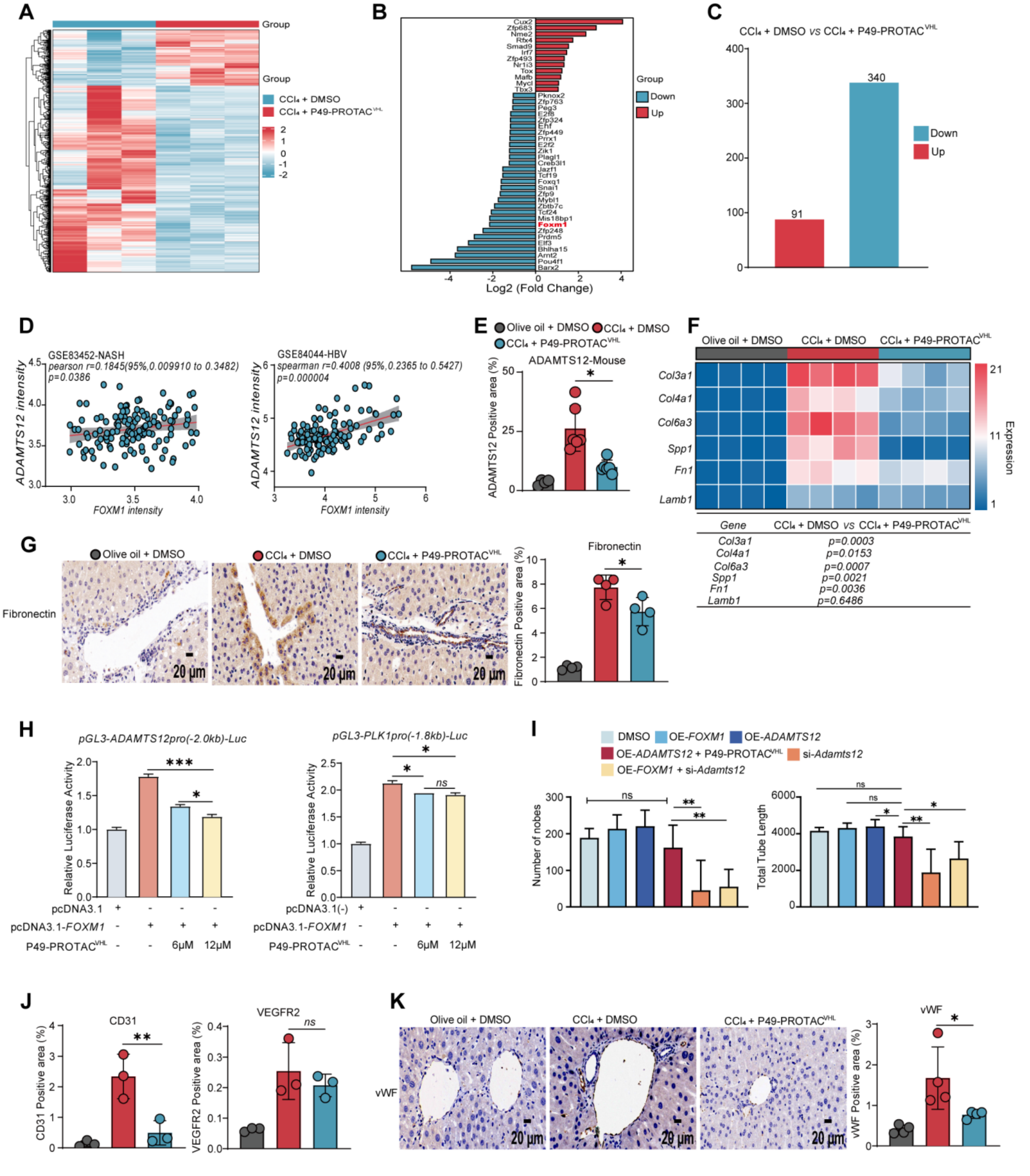

Figure S5. (A) Heatmap of differentially expressed genes in liver tissues from CCl_4 + DMSO and CCl_4 + P49-PROTAC^{VHL} groups (n = 3). **(B)** Transcription factor enrichment analysis. **(C)** Summary of differentially expressed genes. **(D)** Correlation analysis of *FOXM1* and *ADAMTS12* in human fibrosis datasets. **(E)** Quantification of ADAMTS12 positive area corresponding to Figure 5G (n = 6). **(F)** qRT-PCR analysis of ECM-related genes in fibrotic mouse livers (n = 4). **(G)** Immunohistochemistry and quantification of fibronectin in mouse livers (n = 4). **(H)** Dual-luciferase reporter assays of *ADAMTS12* and *PLK1* promoter activity (n = 3). **(I)** Quantification of node number and tube length corresponding to

Figure 5J (n = 4). **(J)** Quantification of CD31- and VEGFR2-positive areas corresponding to Figure 5K (n = 3). **(K)** Immunohistochemistry and quantification of von Willebrand factor (vWF) in mouse livers (n = 4). Data are mean ± SD. *$^{*}P < 0.05$, $^{**}P < 0.01$, $^{***}P < 0.001$*.

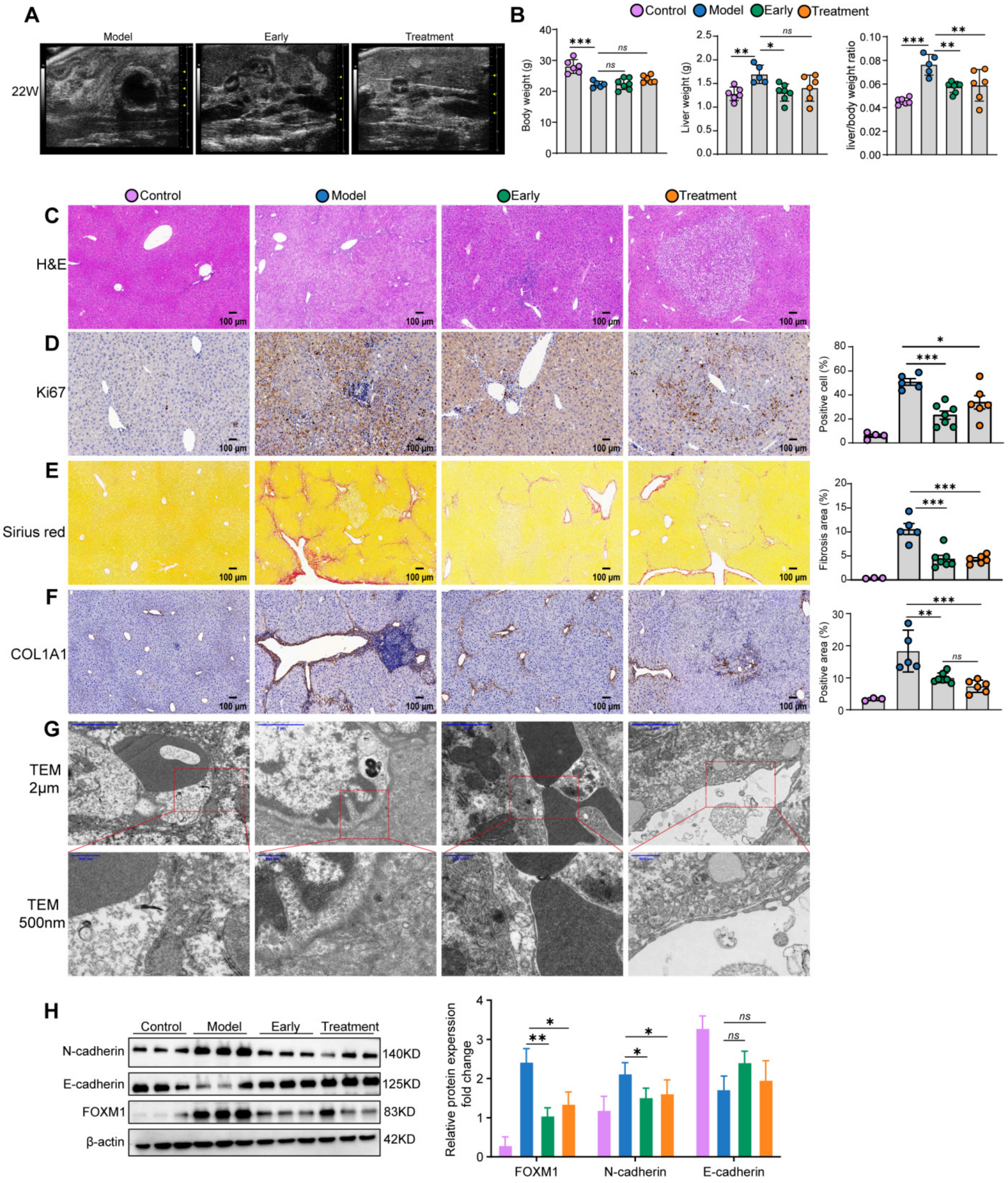

Figure S6. (A) Representative ultrasound images at week 22. **(B)** Body weight, liver weight, and liver index (Control, n = 6; Model, n = 5; Early, n = 7; Treatment, n = 6). **(C)** H&E staining of liver sections. Scale bar, 100 μm. **(D)** IHC staining for Ki-67 and quantification of Ki-67-positive

cells. Scale bar, 100 μm (n = 4). **(E)** Sirius Red staining and quantification of positive area. Scale bar, 100 μm (n = 4). **(F)** IHC staining for COL1A1 and quantification of positive area. Scale bar, 100 μm (n = 4). **(G)** TEM images showing sinusoidal basement membrane morphology. Scale bars, 2 μm and 500 nm. **(H)** Western blot analysis of FOXM1, N-cadherin, and E-cadherin in liver tissues (n = 3). Data are mean ± SD. $*P < 0.05$, $**P < 0.01$, $***P < 0.001$.

Table S1. Antibodies and flow-cytometry reagents

Name	Supplier	Cat no.
FOXM1	Abcam	ab207298
Ubi	Immunoway	YM3636
β-tubulin	ABclonal	A12289
α-SMA	ABclonal	A17910
COL1A1	ABclonal	A1352
ADAMTS12	Proteintech	24934-1-AP
CD31	ABclonal	A19014
Albumin	ABclonal	A1363
CD11b	Servicebio	GB115689
VEGFR2	Proteintech	26415-1-AP
STAB2	Immunoway	YN2255
CD34	ABclonal	A19015
Ki-67	Proteintech	27309-1-AP
GAPDH	ABclonal	A19056
TGF-β1	MCE	HY-P80521
BD Pharmingen Stain Buffer	BD Pharmingen	554656
Rat Anti-Mouse CD16/CD32	BD Pharmingen	553141
7-AAD	BD Pharmingen	559925
FITC Hamster Anti-Mouse CD3e	BD Pharmingen	553061
PE Hamster Anti-Mouse CD11c	BD Pharmingen	557401
PE-Cy7 Rat Anti-CD11b	BD Pharmingen	552850
Alexa Fluor 647 Rat Anti-Mouse I-A/I-E	BD Pharmingen	562367
BV421 Mouse Anti-Mouse NK-1.1	BD Pharmingen	562921
V500 Rat Anti-Mouse CD45	BD Pharmingen	561487
Brilliant Violet 650 anti-mouse F4/80	Biolegend	123149

B220	Servicebio	GB113886
CD3	Servicebio	GB13014
N-cadherin	ABclonal	A0433
E-cadherin	ABclonal	A20798
FEM1C	UpingBio technology Co.,Ltd	YP-mAb-19205
KEAP1	UpingBio technology Co.,Ltd	YP-mAb-04318
CRBN	UpingBio technology Co.,Ltd	YP-mAb-18203
VHL	UpingBio technology Co.,Ltd	YP-mAb-00548

Table S2. Cell lines

**Name**	**Supplier**	**Cat no.**
HCCLM3	Shanghai Cell Bank, Chinese Academy of Sciences	TCHu270
HepG2	Shanghai Cell Bank, Chinese Academy of Sciences	SCSP-510
Huh-7	Shanghai Cell Bank, Chinese Academy of Sciences	TCHu182
HEK293T	Shanghai Cell Bank, Chinese Academy of Sciences	GNHu17
LO-2	Xiamen Immocell Biotechnology	IM-H043
LX-2	Xiamen Immocell Biotechnology	IM-H044
HUVEC	Shanghai Cell Bank, Chinese Academy of Sciences	SCSP-5330

Table S3. Animals

**Name**	**Supplier**	**Strain**	**Sex**	**Age**	**Number**
Mice: CCl_4-induced liver fibrosis	Aniphe Biolaboratory Inc	C57BL/6J	Male	8 weeks	30
Mice: DEN+CCl_4-induced HCC model	Aniphe Biolaboratory Inc	C57BL/6J	Male	14 days	36

Table S4. Sequence-based reagents

**Name**	**Forward Sequence (5' to 3')**	**Reverse Sequence (5' to 3')**
Foxm1-Rat	GCCAATTTCAAACAGCGGAACA	CAAGCTTTGACCCTCACAGG
Gapdh-Rat	ACTCTACCCACGGCAAGTTC	TGGGTTTCCCGTTGATGACC
FOXM1-Human	ACCCAAACCAGCTATGATGCC	TCTCCCGTTTCTGCTCGCAAA

CDC25B-Human	ACGCACCTATCCCTGTCTCG	TTCAAACGTCTGCTCCGCCAT
CCNB1-Human	TGAGAGCCATCCTAATTGACT	AATTATTCTGCATGAACCGAT
GAPDH-Human	GAAACTGTGGCGTGATGGC	CACCACTGACACGTTGGCAG
Foxm1-Mouse	GTCTCCTTCTGGACCATTCACC	GCTCAGGATTGGGTCGTTTCTG
Gapdh-Mouse	CATCACTGCCACCCAGAAGACTG	ATGCCAGTGAGCTTCCCGTTCAG
Adamts12-mouse	GCCTTGACAATGACGTGGAGAAG	CCTTCTTGACACAATGAGCAGCC
Col1a1-mouse	CCTCAGGGTATTGCTGGACAAC	CAGAAGGACCTTGTTTGCCAGG
Acta2-mouse	TGCTGACAGAGGCACCACTGAA	CAGTTGTACGTCCAGAGGCATAG
Tgfβ1-mouse	TGATACGCCTGAGTGGCTGTCT	CACAAGAGCAGTGAGCGCTGAA
Col3a1-mouse	GACCAAAAGGTGATGCTGGACAG	CAAGACCTCGTGCTCCAGTTAG
Col4a1-mouse	ATGGCTTGCCTGGAGAGATAGG	TGGTTGCCCTTTGAGTCCTGGA
Timp-1-mouse	TCTTGGTTCCCTGGCGTACTCT	GTGAGTGTCACTCTCCAGTTTGC
Spp1-mouse	GCTTGGCTTATGGACTGAGGTC	CCTTAGACTCACCGCTCTTCATG
Fn1-mouse	CCCTATCTCTGATACCGTTGTCC	TGCCGCAACTACTGTGATTCGG
Lamb1-mouse	GAACTACACGGTGAGGTTGGAG	GCCAACAGTGAAGATGTCCAGC

Table S5. Target sequences of siRNAs used in this study

Name	Sense Sequence (5' to 3')	Supplier
siNC	Proprietary sequence	Beijing Tsingke Biotech Co., Ltd
siADAMTS12[1]	GCCAAAGUUUGGAGGGAAA	Beijing Tsingke Biotech Co., Ltd

Table S6. *Adamts12* FISH probe sequences

Probe Name	Sequence (5' to 3')
Probe1	5'-ATAAGCTGAGCCACGATCGAGAGC-3'
Probe2	5'-GACAGAACATGGCCACCAGCATCG-3'
Probe3	5'-GGACTAGATTGCCAATGCTTGGGC-3'
Probe4	5'-GAACAGTGTGACCAGGGTGACCAG-3'
Probe5	5'-CAAATTCACTGCACTGCATCTGCC-3'

Table S7. Software

Software name	Manufacturer	Version
GraphPad Prism	GraphPad Software	9.5.1
ModFit LT	Verity Software House	5.0.0
FlowJo	Flexera Software	10.8.1
ImageJ	National Institutes of Health	1.8.0
SnapGene	GSL Biotech	6.0.2
IBM SPSS Statistics	IBM Information Management	23.0.0

Table S8. Reagents

Reagents	Source	Catalog number
Hyaluronic Acid ELISA Kits	Mlbio, Shanghai, China	ml500128
Laminin ELISA Kits	Mlbio, Shanghai, China	ml063190
Procollagen Type III ELISA Kits	Mlbio, Shanghai, China	ml107281
Type IV Collagen ELISA Kits	Biosharp, Beijing, China	BWEM-1090
Hydroxyproline ELISA Kits	Biosharp, Beijing, China	BWEM-928
Superoxide dismutase ELISA Kits	Servicebio, Wuhan, China	G4306
Glutathione ELISA Kits	Servicebio, Wuhan, China	G4305
Malondialdehyde ELISA Kits	Servicebio, Wuhan, China	G4302
Catalase ELISA Kits	Servicebio, Wuhan, China	G4307
TNF-α ELISA Kits	Solarbio, Beijing, China	SEKM-0034
IL-6 ELISA Kits	Solarbio, Beijing, China	SEKM-0007
IL-1β ELISA Kits	Biosharp, Beijing, China	BXEM-722
IL-10 ELISA Kits	Biosharp, Beijing, China	BWEM-274
Mouse Tumor Tissue Dissociation Kit	Absin, Shanghai, China	abs50090
DMEM Medium	Gibco, Thermo Fisher Scientific	11995065
Fetal Bovine Serum	Gibco, Thermo Fisher Scientific	A5256701
Cell Counting Kit-8	MedChemExpress	HY-K0301
Matrigel	Solarbio, Beijing, China	356234

Annexin V-FITC/PI Apoptosis Detection Kit	Vazyme, Nanjing, China	A211
JetPRIME Transfection Kit	Polyplus, France	101000046
RIPA Buffer	Beyotime, Shanghai, China	P0013B
PMSF	Beyotime, Shanghai, China	ST505
ECL Substrate	Beyotime, Shanghai, China	P0018FS
IP Lysis Buffer	ABclonal, Wuhan, China	RM00022
pGL3.1-Basic	Miaoling, Wuhan, China	P0193
pcDNA3.1	Miaoling, Wuhan, China	P0157
FastPure RNA Isolation Kit	Vazyme, Nanjing, China	RC113-01
HiScript IV cDNA Synthesis Kit	Vazyme, Nanjing, China	R412-01
Taq Pro Universal SYBR Green Master Mix	Vazyme, Nanjing, China	Q712-02
Double-Luciferase Reporter Assay Kit	GENE CREATE, Wuhan, China	JKR23008
CCl_4	MedChemExpress	HY-Y0298
Olive Oil	MedChemExpress	HY-108749
Diethylnitrosamine	MedChemExpress	HY-N7434
Masson's Trichrome Kit	Solarbio, Beijing, China	G1340
Modified Sirius Red Stain Kit	Solarbio, Beijing, China	G1472
DAB Substrate Kit	Solarbio, Beijing, China	G1212
RNASweAMI *In Situ* Hybridization CY3 Detection Kit	Servicebio, Wuhan, China	GF002
TSAPLus Fluorescent Six-Marker Seven-Color Staining Kit	Servicebio, Wuhan, China	G1257-50T
Calcein AM	Servicebio, Wuhan, China	G1728
Lipomaster 3000 Transfection Reagent	Vazyme, Nanjing, China	TL301

References

1. Dekky B, Azar F, Bonnier D, Monseur C, Kalebić C, Arpigny E, et al. ADAMTS12 is a stromal modulator in chronic liver disease. FASEB J. 2023; 37: e23237.
